# Two adhesive systems cooperatively regulate axon ensheathment and myelin growth in the CNS

**DOI:** 10.1038/s41467-019-12789-z

**Published:** 2019-10-22

**Authors:** Minou Djannatian, Sebastian Timmler, Martina Arends, Manja Luckner, Marie-Theres Weil, Ioannis Alexopoulos, Nicolas Snaidero, Bettina Schmid, Thomas Misgeld, Wiebke Möbius, Martina Schifferer, Elior Peles, Mikael Simons

**Affiliations:** 1 Institute of Neuronal Cell Biology, Technical University Munich, Munich, Germany; 2 German Center for Neurodegenerative Diseases (DZNE), Munich, Germany; 3 Department of Neurogenetics, Max Planck Institute of Experimental Medicine, 37075 Göttingen, Germany; 4 Center for Nanoscale Microscopy and Molecular Physiology of the Brain (CNMPB), Göttingen, Germany; 5 Munich Cluster of Systems Neurology (SyNergy), Munich, Germany; 6 Electron Microscopy Core Unit, Max Planck Institute of Experimental Medicine, 37075 Göttingen, Germany; 7 Department of Molecular Cell Biology, The Weizmann Institute of Science, Rehovot, Israel; 8 Max Planck Institute of Experimental Medicine, Göttingen, Germany

**Keywords:** Neuroscience, Glial biology, Oligodendrocyte

## Abstract

Central nervous system myelin is a multilayered membrane produced by oligodendrocytes to increase neural processing speed and efficiency, but the molecular mechanisms underlying axonal selection and myelin wrapping are unknown. Here, using combined morphological and molecular analyses in mice and zebrafish, we show that adhesion molecules of the paranodal and the internodal segment work synergistically using overlapping functions to regulate axonal interaction and myelin wrapping. In the absence of these adhesive systems, axonal recognition by myelin is impaired with myelin growing on top of previously myelinated fibers, around neuronal cell bodies and above nodes of Ranvier. In addition, myelin wrapping is disturbed with the leading edge moving away from the axon and in between previously formed layers. These data show how two adhesive systems function together to guide axonal ensheathment and myelin wrapping, and provide a mechanistic understanding of how the spatial organization of myelin is achieved.

## Abstract (web summary)

It remains unclear how myelin is targeted specifically to axons while sparing neuronal cell bodies and dendrites, or how small gaps, the nodes of Ranvier, are left unmyelinated along the axon. In this study, authors used genetic analyses in zebrafish and mice to demonstrate that molecules of the paranodal axo-glial junction act jointly with molecules of the internodal domain to regulate axonal interactions and myelin wrapping, and that in the combined absence of these molecules myelin sheaths are misplaced.

## Introduction

Myelin in the CNS is formed by oligodendrocytes, each of which extends numerous processes to form distinct myelin internode segments along axons, thereby increasing neural processing speed and energetic efficiency^1^. Modulation of myelin structure plays an important role in neuronal circuit behavior, a function which extends into adult life and contributes to brain plasticity and learning^2–11^. Myelin has been studied intensively, not only because of its important role in brain function, but also due to its contribution to diseases such as multiple sclerosis. Yet, our understanding of myelin formation is only starting to advance from a cellular to a molecular level. The observation that oligodendrocytes are able to ensheath and/or wrap myelin around cylindrically shaped substrates of carbon glass or polymer and even micropillars have led to the concept that oligodendrocytes are unselective, and use physical cues such as axon caliber size as one main determinant for myelination^12–14^. However, myelination of inert substrate is not equivalent to the fine-tuned ensheathment of axons within neuronal networks, which requires precise patterning and spatial organization of myelin. For example, myelin sheath length has to be adapted within circuits to precisely control co-incidence of action potential arrival times^15,16^, and nodes of Ranvier have to be generated at the correct axonal position to enable optimal nerve conduction. Thus, molecular mechanisms must exist that determine axon ensheathment, myelin length and thickness.

Adhesion molecules mediate bidirectional interactions between cells and provide specificity for target recognition in brain development, but whether and how they contribute to the highly dynamic process of myelination is not known^17–19^. Myelin growth occurs by the wrapping of the leading edge at the inner tongue around the axon, underneath the previously deposited membrane, together with the lateral extension of all myelin lamellae towards the nodal regions^20^. Thus, for myelin growth, the leading edge must continuously displace myelin from the axon to position newly made membrane layers growing underneath. Strong adhesive forces between the axon and the leading edge of the growing myelin sheath may not be compatible with such a model as they would block the movement around the axon^21–23^. Nevertheless, axo-glial adhesion molecules have been identified in myelinated axons. Some of these molecules are localized at the paranodal and internodal segment (the portion of the axonal/glial membrane located under the compact myelin sheath), among which are the immunoglobulin cell adhesion molecules (Cadm) (i.e., Cadm3/Necl1/SynCAM1 and Cadm4/Necl4/SynCAM4)^24,25^ and the Sialic acid-binding immunoglobulin-type lectins family of proteins (myelin-associated glycoprotein, MAG). These adhesion molecules are ideally positioned to drive myelin growth at the innermost myelin membrane, but the phenotype of the respective knockout mice is subtle with morphologically intact myelin and only minor changes in myelin integrity^26–28^. In addition, axo-glial adhesion molecules at the paranode form a tripartite complex, composed of contactin-associated protein (Caspr) and contactin-1 (Cntn1) at the axonal and neurofascin (Nfasc155) at the glial side^29–32^. Mice lacking the essential components of the paranodal axon-glial junctions suffer from conduction blocks^33,34^, caused by current leakage, but the paranodal adhesion molecules do not appear to be required for myelin growth, as myelin is still generated when the axo-glial junctions are not formed^35–37^.

Here, we hypothesized that axo-glial adhesion molecules work synergistically using overlapping but also specific functions to coordinate axon ensheathment and myelin wrapping. We combined imaging with genetic analyses in mice and zebrafish to obtain a molecular and dynamic model of myelin generation in the CNS. Our data show that adhesion molecules of the paranodal axo-glial junction act jointly with MAG to regulate myelin growth. In their combined absence, myelin is growing on top of each other and above nodes. In addition, myelin layers are misarranged with one leading edge moving around the axon and another moving into the sheath, generating double myelin sheaths. Our data support a model in which these two adhesive systems work together to clamp the leading edge to the axon allowing the extending leading edge to move around the axon without forming promiscuous contacts.

## Results

### Adhesion molecules control myelination in zebrafish

**Fig. 1:**
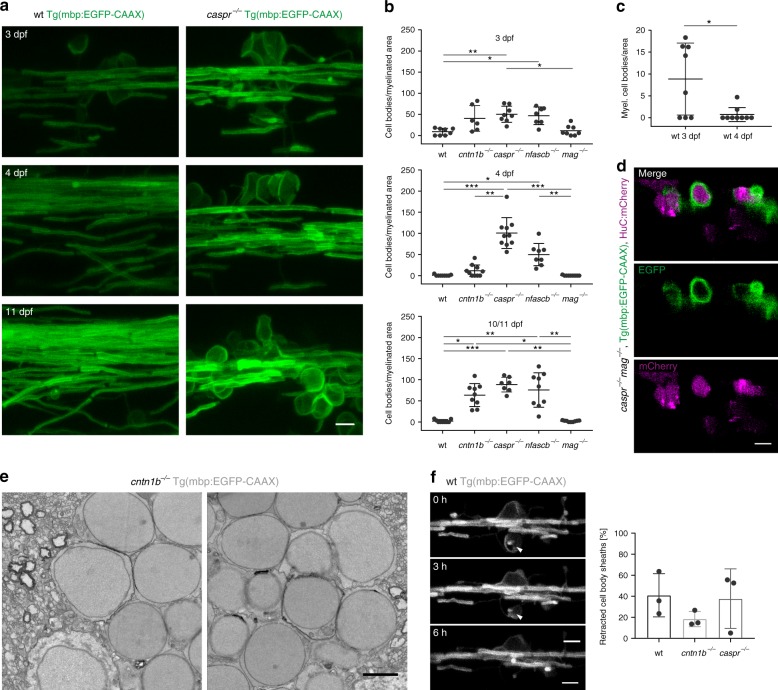
Loss of adhesion molecules of the paranodal axo-glial junction results in neuronal cell body wrapping. **a** CNS myelin of 3, 4 and 10 dpf wild-type (wt) and *caspr*^*−/−*^ fish. Note cell body wrappings in *caspr*^*−/−*^ fish. **b** Cell body wrappings per myelinated area at 3, 4, and 10/11 dpf (*n* = 6–10, Kruskal Wallis ANOVA: 3 dpf: *p* = 0.0004, 4 dpf and 10/11 dpf: *p* < 0.0001). **c** Cell body wrappings in wt fish (*n* = 8–9). Data derived from panel (**b**). Mann-Whitney test, *p = 0.0399. **d** HuC:mCherry expression in 4 dpf *caspr*^*−/−*^*mag*^*−/−*^ fish shows neuronal cell bodies enwrapped by myelin membrane. **e** SEM images of cell body wrappings in *cntn1b*^*−/−*^ fish at dpf 10. **f** Sheath retractions (arrow) from neuronal cell bodies (30 min intervals, 15–16 h time lapse experiments, *n* = 3, Kruskal-Wallis one-way ANOVA: *p* = 0.4393). Images (**a, d, f**) are maximum intensity projections of Tg(mbp:EGFP-CAAX) zebrafish dorsal spinal cord. *p* values, * < 0.05, ** < 0.01, *** < 0.001. Data are presented as means ± s.d.Scale bars, 5 mm (**a, d, f**), 2 µm (**e**). Source data are provided as a Source Data file

We started our analysis of adhesion molecules in myelination using zebrafish as a model system, taking advantage of its genetic tractability and amenability for high-resolution in vivo imaging. Unlike mice, zebrafish have duplicated genes for *cntn1* and *neurofascin*, but not for *caspr* and *mag*, resulting in c*ntn1a* and c*ntn1b*, and *nfasca* and *nfascb* genes^38^. To determine which of the proteins localizes to the axo-glial junction at the paranode in fish, we co-injected plasmids encoding for HuC:Gal4 and UAS:EGFP-cntn1a or UAS:EGFP-cntn1b into Tg(Sox10:mRFP) fish^39^ (Supplementary Fig. 1). We observed that EGFP-cntn1a localized to the node (Supplementary Fig. 1a), as shown previously^40^, whereas EGFP-cntn1b was targeted to paranodes (Supplementary Fig. 1b). Expression of caspr-YFP in neurons and nfascb-EGFP in glia resulted in paranodal distribution (Supplementary Fig. 1c, d), indicating that cntn1b, nfascb and caspr are localized to the axo-glial junction in zebrafish. Whereas the function of the adhesion molecules of the paranodal axo-glial junction in the assembly of paranodes and nodes is well established, their role in myelination is unclear. We used CRISPR/Cas9 based genome editing to target the corresponding genes of the paranodal axo-glial junction and *mag* by injecting single-stranded target-specific gRNAs together with recombinant Cas9 protein into fertilized one-cell stage embryos of Tg(mbp:EGFP-CAAX) fish. Heterozygous *F*1 siblings carrying identical germline mutations were then crossed to generate *F*2 homozygous mutants (Supplementary Fig. 1e), in which gene inactivation was confirmed using real-time PCR of the respective gene products (Supplementary Fig. 1f–i). Whereas *mag* mutants were indistinguishable from wild-type fish, *cntn1b*^*−/−*^, *caspr*^*−/−*^, and *nfascb*^*−/−*^ mutants were infertile, of smaller size (Supplementary Fig. 1e), with ataxic swimming behavior and developed a curved spine with time. The most striking feature observed in all three mutants of the paranodal axo-glial junction, but not *mag*^*−/−*^, was ensheathment/myelination of circular profiles (Fig. 1a, b), representing neuronal cell bodies, as shown by the co-expression of mCherry in neurons and EGFP-CAAX in oligodendrocytes (Fig. 1d). We collected 500 different adjacent 200 nm sections using an automated tape collecting ultramicrotome (ATUMtome) for scanning electron microscopy and found one or more layers of membrane around the cell bodies in *cntn1b*^*−/−*^ mutants (Fig. 1e). In *cntn1b*^*−/−*^, *caspr*^*−/−*^, and *nfascb*^*−/−*^ mutants, cell bodies wrappings appeared at 3 dpf and increased with time, with higher numbers at 4 and 10 dpf as compared to 3 dpf (Fig. 1a, b). Notably, cell body wrappings were also observed in most wild-type fish at 3 dpf, but had mostly disappeared one day later, at 4 dpf (Fig. 1c). Using in vivo time-lapse confocal imaging between 3 and 4 dpf, we confirmed retractions of sheaths from cell bodies in wild-type fish (Fig. 1f and Supplementary Movie 1), but also in *cntn1b*^−/−^, *caspr*^−/−^ fish, suggesting that their formation is caused by increased myelin mistargeting in mutants.

**Fig. 2:**
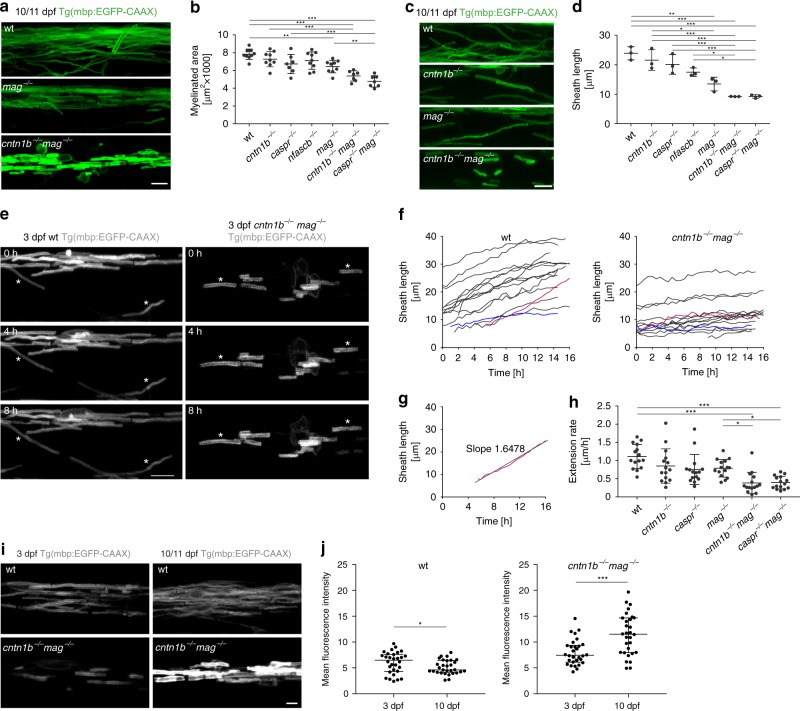
Mag and adhesion molecules of the paranodal axo-glial junction regulate myelin growth in zebrafish. **a** CNS myelin of 10/11dpf wild-type (wt), *mag*^*−/−*^ and *cntn1b*^*−/−*^*mag*^*−/−*^ fish. **b** Myelinated area at 10/11 dpf (*n* = 7–9; one-way ANOVA: *p* < 0.0001). **c** Representative wt and mutant myelin sheaths at 10/11 dpf. **d** Sheath length at 10/11 dpf (means of 60 sheaths per animal, *n* = 3, one-way ANOVA, *p* < 0.0001). **e**–**h** Myelin sheath extension at 3 dpf (30 min interval, 15–16 h time lapse experiments). Asterisks in panel (**e**) represent extending sheaths. Sheath length over time is shown in panel (**f**) (15 sheaths from 3 animals). The fastest (magenta) and slowest (blue) extending sheath are highlighted. Extension rates (**h**) were calculated from (**f**) according to panel (**g**). One-way ANOVA: *p* < 0.0001. **i** Myelin sheaths of 3 dpf and 10 dpf *cntn1b*^*−/−*^*mag*^*−/−*^ show differences in sheath intensity compared to wt. **j** Mean fluorescence intensities of 30 representative sheaths from 3 fish at 3 and 10 dpf, wt (left) and *cntn1b*^*−/−*^*mag*^*−/−*^ (right). Unpaired two-sided t test: *p* = 0.0473 (wt), *p* = 0.0002 (*cntn1b*^*−/−*^*mag*^*−/−*^). Data are presented as means ± s.d. Scale bars, 10 μm (**a, c, e**), 5 μm (**i**). Source data are provided as a Source Data file

Next, we analyzed the extent of myelin formation in *mag*^*−/−*^, *cntn1b*^*−/−*^, *caspr*^*−/−*^ and *nfascb*^*−/−*^ mutants, and also generated double mutants to determine additive effects. Surprisingly, combining *mag*^*−/−*^ with *cntn1b*^*−/−*^ or *caspr*^*−/−*^ mutants resulted in a less severe phenotype than single *cntn1b*^*−/−*^ and *caspr*^*−/−*^ mutants with almost normal body posture and only minor ataxic swim pattern, although these fish remained significantly smaller compared to wild-types (Supplementary Fig. 1e). Despite the milder phenotype, *cntn1b*^*−/−*^*mag*^*−/−*^ and *caspr*^*−/−*^*mag*^*−/−*^ double mutants were more severely hypomyelinated compared to the single mutants as determined by light and electron microscopy (Fig. 2a, b and Supplementary Fig. 2a, b). To elucidate the observed hypomyelination in double mutants, we quantified the number of oligodendrocytes, the number of sheaths formed per oligodendrocytes and the sheath lengths. Whereas the number of oligodendrocytes did not differ between mutants and wild-type (Supplementary Fig. 2c), we found that double mutants formed fewer myelin sheaths per oligodendrocytes (Supplementary Fig. 2d, e). Cell body wrappings were also detected in *cntn1b*^*−/−*^*mag*^*−/−*^ and *caspr*^*−/−*^*mag*^*−/−*^ double mutants (Supplementary Fig. 2f, g). One of the most striking differences was the reduction of myelin sheath length in double mutants (9.2 ± 4.9 µm in *cntn1b*^*−/−*^*mag*^*−/−*^ and 9.3 ± 4.6 µm in *caspr*^*−/−*^*mag*^*−/−*^ vs. 17.1 ± 8.5 µm in wild-type fish, error values represent s.d., Supplementary Fig. 2h, i). This effect cannot be explained by a delay in the onset of myelination, as the sheaths remained short even at 10 dpf (10.2 ± 5.8 µm in *cntn1b*^*−/−*^*mag*^*−/−*^ and 9.6 ± 5.0 µm in *caspr*^*−/−*^*mag*^*−/−*^ vs. 23.9 ± 10.6 µm in wild-type fish, Fig. 2c, d). We also analyzed *cntn1b*^*−/−*^*nfascb*^*−/−*^ double mutants and found that myelin sheaths lengths were similar to single mutants and only an increase in cell body wrappings was observed (Supplementary Fig. 2j–m).

### Myelin sheath elongation and actin dynamics require adhesion

**Fig. 3:**
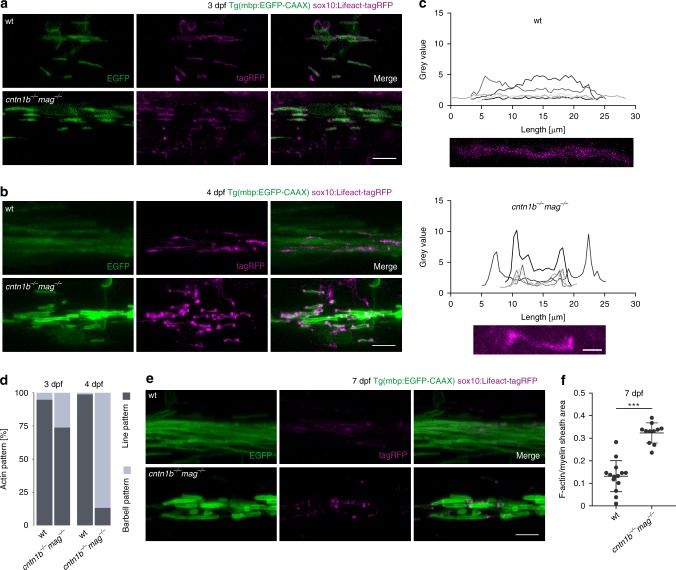
Loss of adhesion molecules results in aberrant F-Actin distribution along the myelin sheath in zebrafish. **a** Myelin sheaths in 3 dpf wild-type (wt) and *cntn1b*^*−/−*^*mag*^*−/−*^ fish expressing Lifeact-tagRFP to label F-actin. Lifeact-tagRFP displayed a line pattern distribution in both wt and mutant. **b** Myelin sheaths in 4 dpf wt and *cntn1b*^*−/−*^*mag*^*−/−*^ fish expressing Lifeact-tagRFP. Lifeact-tagRFP displayed a line pattern distribution in wt and a barbell pattern in mutants. **c** Representative images and quantification of Lifeact-tagRFP fluorescence intensities along example sheaths at 4 dpf. **d** Ratio of barbell-shaped and line-shaped patterns in myelin sheaths (*n* = 3–5) at 3 and 4 dpf. **e** Myelin sheaths in7 dpf wt and *cntn1b*^*−/−*^*mag*^*−/−*^ fish expressing Lifeact-tagRFP. Lifeact-tagRFP had almost disappeared in wt, whereas the barbell pattern persisted in mutants. **f** Quantification of the area occupied by F-actin within myelin sheaths at 7 dpf in wt and *cntn1b*^*−/−*^*mag*^*−/−*^ fish (*n* = 11–14). Images (**a, b, e**) are maximum intensity projections of Tg(mbp:EGFP-CAAX) zebrafish dorsal spinal cord. *p* values, *** < 0.001. Data are presented as means ± s.d. Scale bars, 10 μm (**a, b, e**), 2 μm (**c**). Source data are provided as a Source Data file

As these results pointed to the role of two adhesive systems in regulating myelination, we performed time-lapse imaging to follow the growth of individual sheaths in Tg(mbp:EGFP-CAAX) mutant fish (Fig. 2e–h). We observed striking differences in myelin sheath elongation rates. Whereas myelin sheaths continuously extended at a rate of 1.1 ± 0.33 µm/h within a time frame of 16 h in wild-type fish, the extension rate dropped to 0.38 ± 0.29 µm/h in *cntn1b*^*−/−*^*mag*^*−/−*^ and 0.39 ± 0.17 µm/h in *caspr*^*−/−*^*mag*^*−/−*^ double mutants (Fig. 2h). In addition, prominent differences in the EGFP-CAAX fluorescence pattern were detected in myelin sheaths of *cntn1b*^*−/−*^*mag*^*−/−*^ and *caspr*^*−/−*^*mag*^*−/−*^ double mutants (Fig. 2i, j and Supplementary Movie 2). As myelin growth is driven by actin filament turnover at the leading edge^21,22^, we investigated its role in myelin wrapping by expressing Lifeact (fused with the red fluorescent protein tag-RFP under the control of sox10 upstream regulatory sequences) in oligodendrocytes to visualize F-actin (Fig. 3). During myelin sheath elongation, F-actin is localized in a thin spiral presumably at the leading edge along individual sheaths^22^. We characterized the different stages of Lifeact-RFP distribution during myelin formation. In wild-type fish, Lifeact-RFP forms a thin spiral line along the internode during myelin sheath elongation and largely disappears from the sheaths when sheath elongation is terminated (Fig. 3a–c, e). When Lifeact-RFP was expressed in oligodendrocytes of *cntn1b*^*−/−*^*mag*^*−/−*^ mutants, a distinct pattern was observed. At 3 dpf, we found that Lifeact-RFP was localized in most sheaths in a pattern as observed in wild-type (Fig. 3a, d), but already one day later, at 4 dpf, Lifeact-RFP displayed a barbell-shaped distribution with accumulation at the lateral edges of the sheaths (Fig. 3b–d). Accumulation of Lifeact-RFP in the barbell-shaped distribution persisted at 7 dpf, the time point at which Lifeact-RFP was largely absent from myelin sheaths in wild-type fish (Fig. 3e). We determined the area occupied by Lifeact-RFP within the myelin sheath (EGFP-CAAX) and find that mutants accumulate increased amounts of F-actin at 7 dpf (Fig. 3f). Together, these results point to impaired myelin wrapping with mislocalized F-actin-rich lateral edges of myelin layers within the elongating myelin sheath.

### Loss of adhesion results in myelin overgrowth in zebrafish

**Fig. 4:**
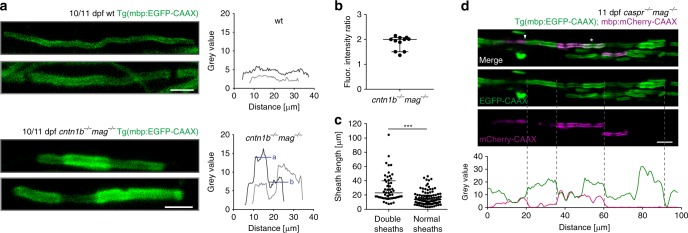
Loss of adhesive molecules results in myelin overgrowth in zebrafish. **a** Myelin sheaths in 10/11 dpf *cntn1b*^*−/−*^*mag*^*−/−*^ fish show fluorescence intensity steps absent in wild-type fish. Fluorescence intensities along example sheaths. ‘a’ and ‘b’ represent mean fluorescence intensity levels. **b** Fluorescence intensity ratios (calculated ‘a’/’b’ according to panel (**a**) in 11 representative *cntn1b*^*−/−*^*mag*^*−/−*^ sheaths (*n* = 4) show two fold increase in fluorescence along the sheath in bright areas as compared to the neighboring regions. Median with interquartile range. **c** Length of sheaths with fluorescence intensity steps (*n* = 53 sheaths from 3 fish) vs. evenly labeled sheaths (*n* = 100 sheaths from 3 fish) in 10 dpf *cntn1b*^*−/−*^*mag*^*−/−*^ fish (two-tailed Mann Whitney test, ***p < 0.0001, median with interquartile range). Sheaths were quantified from a more dorsal region compared to Fig. 2. **d** Expression of mbp:mCherry-CAAX in 11 dpf *caspr*^*−/−*^*mag*^*−/−*^ fish reveals double myelin sheaths (asterisk) and loss of nodes of Ranvier (arrowhead). Fluorescence intensity plot along the upper myelinated axon (green = EGFP-CAAX, magenta = mCherry-CAAX). Dotted lines represent corresponding positions in image and plot. Images (**a, d**) are maximum intensity projections of Tg(mbp:EGFP-CAAX) zebrafish dorsal spinal cord. Scale bars, 4 μm (**a**), 10 μm (**d**). Source data are provided as a Source Data file

When following the EGFP-CAAX fluorescence pattern of myelin sheaths in *cntn1b*^*−/−*^*mag*^*−/−*^ and *caspr*^*−/−*^*mag*^*−/−*^ mutants, we noted a striking step-like increase in fluorescence intensity along some of the sheaths (Fig. 4a). The rise in fluorescence intensity was about two fold (Fig. 4b) and accompanied by a focal increase in sheath thickness. Such sheaths were in average longer compared to sheaths displaying even fluorescence intensities (Fig. 4c), suggesting that two sheaths had grown on top of and into each other. Such patterns of double myelin sheaths were rarely found in *cntn1b*^*−/−*^_,_ *nfascb*^*−/−*^, or *caspr*^*−/−*^ single mutants (Supplementary Fig. 3a). To determine whether these myelin profiles represent, indeed, double myelinated segments, we injected a plasmid encoding for mbp:mCherry-CAAX into *caspr*^*−/−*^*mag*^*−/−*^Tg(mbp:EGFP-CAAX) fish (Fig. 4d). This resulted in the visualization of sparsely mCherry-CAAX-labeled myelin sheaths in Tg(mbp:EGFP-CAAX) fish. Inspection of fluorescence pattern revealed that EGFP-CAAX labeled on top of mCherry-CAAX labeled myelin sheaths. Such ectopic myelination could be a result of a failure of myelin targeting and/or retraction to correct for myelin inappropriately generated around axons^7,8,17,41,42^. We determined retraction rates by time lapse imaging, but found only differences in the retraction speed in *cntn1b*^*−/−*^*mag*^*−/−*^ and *caspr*^*−/−*^*mag*^*−/−*^ mutants (Supplementary Fig. 3b-e and Supplementary Movie 3). Thus, in the combined absence of two adhesive systems, myelin targeting and dynamics are severely impaired resulting in shorter and misplaced myelin segments.

### Adhesion molecules regulate myelination in mice

To determine whether these findings could be recapitulated in a mammalian system, we crossbred a number of different mouse lines deficient in axo-glial adhesion molecules (*Cntn1*^*−/−*^, *Caspr*^*−/−*^*, Caspr2*^*−/−*^ and *Mag*^*−/−*^ mice). While *Cntn1*^*−/−*^ mice developed severe locomotor abnormalities and failure to thrive until P12, *Cntn1*^*−/−*^*Mag*^*−/−*^ mice displayed a somewhat milder phenotype, being slightly heavier and less paralyzed, similar to what we have observed in zebrafish (Supplementary Fig. 4a–c). We used transmission electron microscopy of cross-sections of the optic nerve and spinal cord and started our analysis by quantifying the amount of myelin in *Cntn1*^*−/−*^, *Mag*^*−/−*^ and *Cntn1*^*−/−*^*Mag*^*−/−*^ mice at post-natal day 12 (P12) (Supplementary Fig. 5a–c). We observed a slight decrease in myelinated axon number in both the spinal cord and the optic nerve of *Cntn1*^*−/−*^ and *Mag*^*−/−*^ mice, as expected^26,27,35^. When double *Cntn1*^*−/−*^*Mag*^*−/−*^ mice were analyzed, we found a much more pronounced reduction of myelinated axons with a decline to 63.9 ± 6.7% in the spinal cord and to 68.6 ± 6.1% in the optic nerve (Supplementary Fig. 5a–c). *Cntn1*^*−/−*^ mice had already reached their humane end point at ~P12, and could therefore not be analyzed later. The less severe phenotype of *Cntn1*^*−/−*^*Mag*^*−/−*^ mice allowed further analysis at P15-16 (possibly because the reduced generation of myelin attenuates the conduction block of *Cntn1*^*−/−*^ mice). Myelin generation continued in *Cntn1*^*−/−*^*Mag*^*−/−*^ mice, but axons remained partially unmyelinated (Supplementary Fig. 5a, d). Double *Cntn1*^*−/−*^*Mag*^*−/−*^ mice also displayed higher g-ratios (the ratio of the inner axonal diameter to the total outer diameter, Supplementary Fig. 5e, f). To determine whether hypomyelination reflected changes in oligodendrocyte number, we carried out immunohistochemistry in the spinal cord, which revealed similar numbers of mature CC1^+^ oligodendrocytes in *Mag*^*−/−*^, *Cntn1*^*−/−*^*Mag*^*−/−*^ and control mice (Supplementary Fig. 5g, h). As observed in zebrafish, quantification of internodal lengths showed that *Cntn1*^*−/−*^*Mag*^*−/−*^ mice formed shorter myelin segments as compared to control and single mutants (Supplementary Fig. 5i, j). We also included Caspr and Caspr2 into our analysis, the latter being required for the formation of axo-glial contacts at juxtaparanodes by its interaction with Tag1/Cntn2^43,44^. We performed electron microscopy analyses of optic nerves at P21 to quantify the amount of myelin in *Mag*^*−/−*^, *Caspr*^*−/−*^, *Caspr*^*−/−*^*Mag*^*−/−*^ and *Caspr*^*−/−*^*Caspr2*^*−/−*^*Mag*^*−/−*^ mice and found a decrease of myelinated axon number in *Caspr*^*−/−*^*Mag*^*−/−*^ and *Caspr*^*−/−*^*Caspr2*^*−/−*^*Mag*^*−/−*^ mice (Supplementary Fig. 5k, l). Cell body wrappings were not observed in *Cntn1*^*−/−*^ or *Mag*^*−/−*^ mice (Supplementary Fig. 6).

### Double myelin sheaths in adhesion molecule-deficient mice

**Fig. 5:**
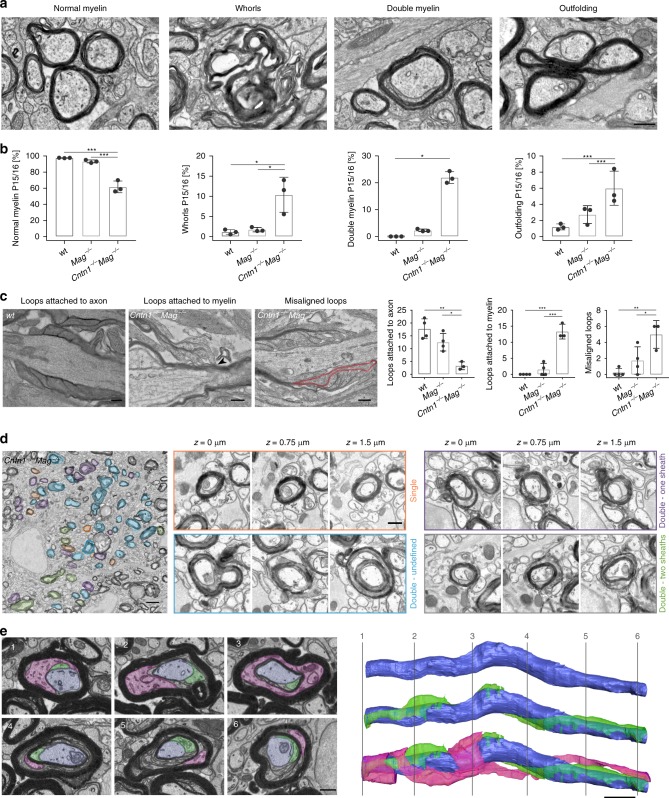
Internodal and paranodal adhesion are required for axonal attachment and correct myelin wrapping in mice. **a**, **b** Electron micrographs (**a**) and quantification (**b**) of myelin and its pathologies in mutants (P15/16 optic nerve cross-section). *n* = 3 mice, one-way ANOVA: *p* < 0.001 (normal myelin), *p* = 0.0076 (whorls), *p* < 0.0001 (double myelin), *p* = 0.0147 (outfoldings)). **c** Electron micrographs and quantification of normal and aberrant paranodes in P12 optic nerves longitudinal sections. Arrow: loop attached to myelin, red outline: misaligned loops. *n* = 3–4 mice, one-way ANOVA: *p* < 0.0013 (loops attached to axon), *p* < 0.0001 (loops attached to myelin), *p* = 0.0063 (misaligned loops)). **d** Automated tape-collection ultramicrotome scanning electron microscopy (ATUM-SEM) image of a P15 *Cntn1*^−/−^*Mag*^−/−^ optic nerve (55 × 55 × 55.05 μm volume) and representative cross-sections. Pseudo-colors indicate normal myelin (orange), double myelin originating from the same myelin sheath (purple), double myelin originating from a second sheath (green), and aberrant myelin that could not be classified (blue). **e** Serial block-face SEM (FIB-SEM) of a P15 *Cntn1*^−/−^*Mag*^−/−^ optic nerve (22 × 15 × 18 µm volume). Cross-sections at different *z*-levels (left) show two separate leading edges of the same myelin sheath: one (green) is attached to the axon (blue), whereas the other one (magenta) is found in between myelin layers. 3D reconstruction (right) shows the axon alone (top), the axon with one (middle) and both leading edges (bottom). Numbering refers to cross-sections. *p* values, * < 0.05, ** < 0.01, *** < 0.001. Data are presented as means ± s.d. Scale bars, 200 nm (**c**), 500 nm (**a, d** (details) and **e** (cross-sections)), 2 μm (**d** (overview) and **e** (reconstruction)). Source data are provided as a Source Data file

When we used transmission electron microscopy of cross-sections of the optic nerve and spinal cord to analyze myelin ultrastructure in *Cntn1*^*−/−*^*Mag*^*−/−*^ in detail, we found that myelin, which was formed, was often with pathological features (Fig. 5a, b and Supplementary Fig. 7a). At P12, 14.2 ± 2.1% of the myelin was abnormal (myelin whorls without associated axon), 12.9 ± 4.7% had outfoldings and 20.1 ± 5.3% represented double myelin (Supplementary Fig. 7a). As double myelinated axons appeared to be the most dramatic feature of *Cntn1*^*−/−*^*Mag*^*−/−*^ animals, we explored the underlying mechanisms by analyzing ultrathin longitudinal section of P15/P16 optic nerves, when myelin can be captured at all stages of growth with a minimal amount of mature sheaths (Fig. 5c). Individual myelin layers were discernible over an average of ~10 µm (77–91 myelination events from three to six different animals per genotype). On longitudinal sections of the myelin sheaths, the lateral edges of each individual myelin layers appeared as cytoplasmic-rich loops attached to the axon in a regular distance in wild-type mice as described previously^20^. When *Cntn1*^*−/−*^*Mag*^*−/−*^ animals were analyzed, we found that the cytoplasmic-rich loops of the individual layers were disorganized. Out of 77 myelin segments that we could follow, a large fraction of the cytoplasmic-rich loops were not in contact with the axons, and often on top of compacted myelin layers (Fig. 5c). In addition, individual loops were frequently misaligned moving in between each other (Fig. 5c). To determine whether some of these double myelin sheaths represented two separate sheaths growing on top of each other, we explored myelin in three dimensions by collecting 150 nm sequential sections using an automated tape collecting ultramicrotome (ATUMtome) for high resolution scanning electron microscopy (Fig. 5d). This allowed us to image a volume of ~55 μm^3^ with a lateral resolution of 4 nm and a *z-*resolution of 150 nm. Within this volume, we analyzed 50 different myelinated axons, of which 26 could be followed in-depth and among those we identified 8 myelin segments consisting of two separate sheaths. Such double myelinated axons were identified by the appearance of paranodal loops arising from a sheath and attaching on top of another sheath.

To elucidate other possible reasons for the double myelinated axons, we performed focused-ion beam scanning electron microscopy (FIB-SEM) of optic nerves. We imaged a volume of ~20 µm^3^ with a lateral resolution of 5 nm and a *z*-resolution of 20 nm, in which we identified and reconstructed 25 myelin sheaths. Surprisingly, we did not only observe double myelin sheaths generated by two separate sheaths growing on top of each other (as shown in a reconstruction in Supplementary Fig. 7b and Supplementary Movie 4), but also double myelin sheaths that seem to arise from one single myelin sheath. We have previously shown that the leading edge (or inner tongue) is closely connected to the axon while moving around the axon to form new layers^20^. However, when the position of the leading edge was analyzed in *Cntn1*^*−/−*^*Mag*^*−/−*^ animals, we found that the leading edge did not remain in contact with the axon in 8 of 25 analyzed sheaths, but was positioned on top of a retained cytoplasmic-rich lateral edge (Fig. 5e). Three dimensional reconstruction of the adjacent sections revealed that there were two leading edges. One remained in contact with the axon, whereas the other one moved away from the axon into the sheath (Supplementary Movie 5). Thus, double myelin sheaths also appear to be generated by a misguided leading edge, moving in two directions—one moving around the axon and one into the sheath—thereby, generating double myelin with two separated regions of compacted myelin layers.

**Fig. 6:**
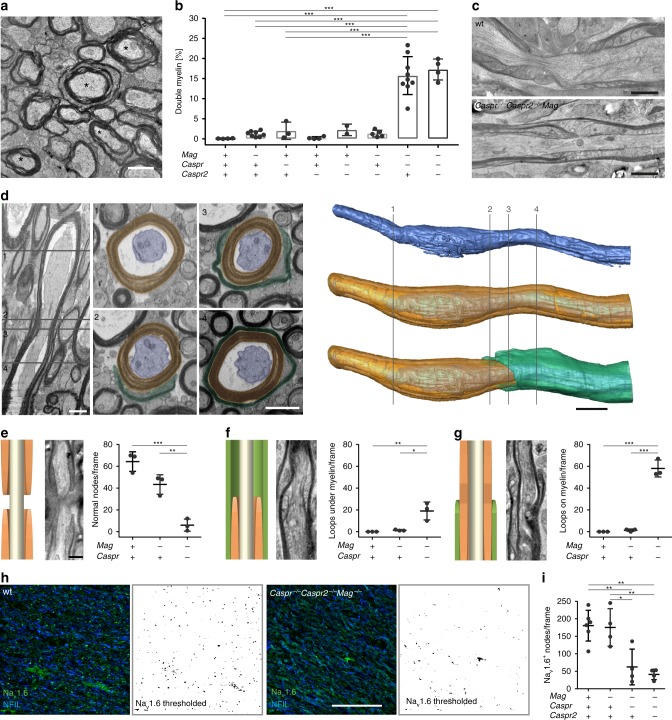
Loss of internodal and paranodal adhesion results in overgrowth of nodes of Ranvier. **a** Electron micrograph of a P21 *Caspr*^*−/−*^*Caspr2*^*−/−*^*Mag*^*−/−*^ mouse optic nerve cross-section. Asterisks indicate double myelin sheaths. **b** Double myelin sheaths in optic nerve cross-sections of P21/P22 mice, 10 frames (219 µm^2^) analyzed per animal (*n* = 2–9; one-way ANOVA: *p* < 0.0001). **c** Electron micrographs of optic nerve longitudinal sections of P21/P22 wt and *Caspr*^*−/−*^*Caspr2*^*−/−*^*Mag*^*−/−*^ mice. **d** Serial block-face SEM (FIB-SEM) of a P21 *Caspr*^−/−^*Caspr2*^−/−^*Mag*^−/−^ optic nerve (13 × 6 × 35 µm), Longitudinal view and cross-sections at different *z*-levels (left): A second myelin sheath (green) encloses an axon (blue) that is already myelinated (orange). 3D reconstruction (right) shows the axon alone (top), compacted myelin around the axon (middle) and an additional myelin sheath on top (bottom). Numbering refers to cross-sections. **e**–**g** Model, electron micrograph and quantification of normal nodes (**e**), loops under myelin (**f**) and loops on top of myelin (**g**) in a 26.000 µm^2^ hexagon grid (*n* = 3). One-way ANOVA: *p* < 0.001 (normal nodes and loops on myelin), *p* < 0.005 (loops under myelin). **h** Optic nerve longitudinal sections from wild-type (wt) and *Caspr*^−/−^*Caspr2*^−/−^*Mag*^−/−^ mice immunostained for Na_v_1.6^+^ sodium channels and pan-neurofilament. **i** Number of Na_v_1.6^+^ sodium channels in wt and mutant animals (*n* = 4–6, one-way ANOVA: *p* < 0.0001). *p* values, * < 0.05, ** < 0.01, *** < 0.001. Data are presented as means ± s.d. Scale bars, 500 nm (**c, e, f, g**), 1 μm (**a, d** (2D sections)), 2 μm (**d** (3D reconstruction)), 50 μm (**h**). Source data are provided as a Source Data file

Next, we included *Caspr*^*−/−*^*Mag*^*−/−*^ and *Caspr*^*−/−*^*Caspr2*^*−/−*^*Mag*^*−/−*^ mice into the analysis, and found that double myelinated sheaths, as detected in *Cntn1*^*−/−*^*Mag*^*−/−*^ mice, was the most striking feature (Fig. 6a–c). We performed FIB-SEM and reconstructed 26 double myelin sheaths from three independent experiments (Fig. 6d and Supplementary Movie 6). We found that of the 26 double myelin sheaths, 13 were generated by two sheaths forming on top of each other, while 5 arose from one single myelin sheath (Supplementary Fig. 7c and Supplementary Movie 7). Collectively, these data from two set of mutants show that double myelinated axons are formed by two mechanisms: i) two myelin sheaths growing on top of each other; and ii) one myelin sheath with two leading edges, one moving around the axon and another moving into the sheath. We also analyzed cross-sections of peripheral sciatic nerves of *Caspr*^*−/−*^*Mag*^*−/−*^ mutant mice by electron microscopy to determine whether pathological profiles were also found in the peripheral nervous system. We did not detect differences in the number of double myelin sheaths in *Caspr*^*−/−*^*Mag*^*−/−*^ and control (Supplementary Fig. 7d, e), demonstrating that function of adhesion molecules differs between the peripheral and central nervous system.

### Overgrowth of nodes of Ranvier in *Mag*^*−/−*^*Caspr*^*−/−*^-mice

The nodal complex initially assembles at the edge of a forming sheath as a heminode, followed by the fusion of two heminodes when two myelin segments elongate and meet each other. For nodes to function, the lateral edges of two neighboring myelin sheaths need to stop growing when they have come into close contact. We asked whether myelin was able to terminate growth at the nodes in *Caspr*^*−/−*^*Mag*^*−/−*^ mice. We investigated the nodes by analyzing ultrathin longitudinal sections of P21 high-pressure frozen optic nerves, when most of the nodes have been generated in wild-type mice (Fig. 6c, e–g). Intact nodes were frequently found in wild-type animals (64.3 ± 9, Fig. 6e). However, when *Caspr*^−/−^*Mag*^−/−^ mice were analyzed, we rarely recognized regions in which two neighboring myelin internodes met and formed a nodal gap (6.0 ± 5.6, Fig. 6e). Instead, we observed that myelin sheaths grew on top or into neighboring sheaths at internode endings. Quantifications revealed aberrant myelin structures resembling paranodal loops under myelin or on top of myelin in *Caspr*^−/−^*Mag*^−/−^ mice (Fig. 6f, g; under myelin: 0 ± 0 (wt) vs. 19.0 ± 8.2 (*Caspr*^−/−^*Mag*^−/−^); on myelin: 0 ± 0 (wt) vs. 58 ± 7.8 (*Caspr*^−/−^*Mag*^−/−^)). We performed immunohistochemistry of P21 spinal cord cross-sections to validate these findings. Na^+^-channel immunolabeling confirmed the loss of the nodal domain in *Caspr*^−/−^*Mag*^−/−^ and *Caspr*^−/−^*Caspr2*^−/−^*Mag*^−/−^ mice (Fig. 6h, i).

## Discussion

Adhesion molecules play a key role in target recognition and the acquisition of positional identity in the central nervous system. While cell–cell adhesions are widely used to generate specific connections in neuronal development, the association of myelin to axons provides a particular challenge to the function of adhesive systems. Myelination is a dynamic process in which the oligodendroglial process, after connecting to a target axon, continuously moves around the axon and thereby constantly forms and breaks adhesive contacts with the axonal surface. While the myelin sheath extends laterally, the lateral edges of the previously formed layers need to be in continuous contact with the axon until they have reached their final position and form the paranodal junctions. We reasoned that such an orchestrated process requires the coordinated actions of different adhesion molecules.

**Fig. 7:**
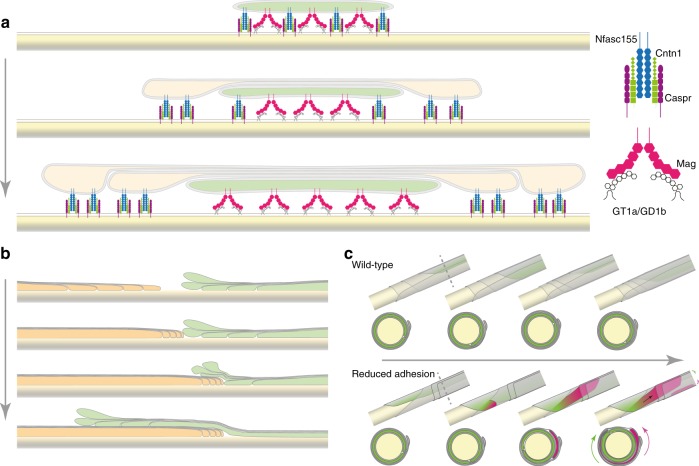
Model of the role of adhesion molecules in myelin wrapping. **a** Lateral model of the localization of adhesion molecule complexes during myelination. The leading edge (green) attaches to the axon (yellow) through adhesion molecules on axon and myelin. While new layers form (top to bottom), previously built layers extend laterally (orange) and stay tightly attached to the axon. **b** Lateral model of two myelin segments (green and orange) growing into each other when internodal and paranodal adhesion is reduced (top to bottom). **c** 3D model and cross-sections of a myelinated axon with intact adhesion (top row) and reduced internodal and paranodal adhesion (bottom row) during myelination (left to right). The leading edge (green) wraps around the axon (yellow). With reduced adhesion, a second leading edge (magenta) moves in between myelin layers and continues wrapping in opposite direction to the first leading edge (arrows). Dotted line indicates position of cross-sections

Using a combination of in vivo imaging and ultrastructural analysis in genetic zebrafish and mouse models, we were able to show that MAG and the paranodal adhesion molecules work together to guide myelin sheath growth. Only in their combined absence, myelin growth is unrestricted, encroaching on top of each other and overgrowing nodes. In addition, myelin wrapping is impaired with the leading edge partially losing its contact to the axon, in such a way that double myelin sheaths are formed. Together, these data led to a model of how adhesion molecules may operate in myelin membrane wrapping and growth, which is summarized in Fig. 7: We suggest that two adhesive systems work together to coordinate the wrapping of the leading edge at the inner tongue around the axon, underneath the previously deposited membrane. Our model implies that the lateral cytoplasm-rich membranous pockets of each myelin layer are kept in close contact with the axonal surface by the function of the paranodal adhesion molecules. While these cytoplasm-rich lateral edges move towards the future node, where they align and position as paranodal loops, they provide a shelter for the movement of the inner tongue (Fig. 7). We believe that this is necessary as the leading edge of the growing myelin sheath needs to move multiple times around the axon with low adhesiveness^21,22^. Thus, one function of the axoglial paranodal adhesion molecules could be to protect the promiscuous leading edge from targeting surfaces unselectively. We propose that myelin is targeted to and expands around the axon in progressive stages: during the interaction of oligodendroglial processes with appropriate axons, MAG binds to gangliosides on the axonal surface and these interactions may displace the axoglial paranodal adhesion molecules to the lateral edges of the developing sheath. While the paranodal adhesion molecules remain tightly associated to each other, the interactions of MAG with axonal gangliosides are constantly renewed. In the absence of only the paranodal adhesion molecules, the interaction of MAG with the axons appears to be sufficient to guide the movement of the inner tongue around the axon. Likewise, if only *Mag* is deleted, the specific targeting of the inner tongue to the axon is functioning with only very few axons being surrounded by two or more myelin sheaths^23,26,27^. However, the combined deficiency of the paranodal adhesion molecules and *Mag*, results in severe disturbances by disrupting both mechanisms that control directed growth of the myelin membrane. Now, the leading edge has not only lost its fixation to the axon, but also its boundaries that keep it clamped to the interior of the myelin sheath. As a result, the leading edge may move out of the myelin sheath, across or beneath another sheath, and on top of nodes. In addition, as the lateral edges of the extending myelin layers fall back, the leading edge moves in some cases on top of previously formed layers resulting in the formation of double myelinated axons. One limitation of our work is that that we had to combine live-imaging and electron microscopy to image myelination, as there are currently no techniques available to directly visualize the movement of individual myelin layers in real time during myelination. Thus, we have to rely on 3D electron microscopy to determine myelin ultrastructure in static images.

One important step for myelination to occur is the segregation of the paranodal adhesion molecules to the lateral edges of the growing myelin sheath. We hypothesize that the segregation of the tightly assembled paranodal adhesion molecules, from the innermost layer of myelin to the lateral edges is important to reduce adhesiveness of the inner tongue. Consistent with this model, excessive axo-glial contacts formed in transgenic mice with increased expression of Cadm4 inhibits myelin growth by the stabilization of otherwise transient or weak interactions^23^. It is important to note that MAG is not only present at the internodal segment, but also at the paranodal junction, it is therefore not clear how and where MAG exerts its adhesive functions to prevent unrestricted myelin growth. One possibility is that MAG is required at the internode to fix the leading edge onto the axon; another is that MAG is essential for the stabilization of the paranodal axo-glial junction.

How do myelin sheaths stop when they have reached the future nodal domain? It is conceivable that while moving laterally, the paranodal complex gradually matures, forming larger protein arrays by the association with several layers of cytoskeleton components consisting of F-actin, spectrin and ankyrins^45^. The lateral interconnection of the complex and its attachment to the submembranous cytoskeleton may generate mechanical resistance that the machinery driving myelin elongation is unable to surmount. Currently, we do not fully understand the molecular details of the force generating apparatus that is necessary for the leading edge to crawl underneath and towards the lateral edges of the forming myelin sheath. One important mechanism seems to be actin dynamics by supporting the iterative cycles of polymerization/depolymerization at the leading edge^21,22^. Adhesion molecules may not only provide structural support for the growth process, but could play a more active role. In general, cells use the lamellipodial principle to move forward based on F-actin polymerization at the cell front that pushes out a membrane protrusion which subsequently becomes anchored to the substrate by transmembrane adhesion receptors. These receptors are often dynamically coupled to the actin cytoskeleton in order to transduce the internal forces that are generated by actomyosin contractility and actin polymerization. One possibility is therefore that these adhesion molecules also support the protrusion of the leading edge by providing adhesive contacts to generate friction for the movement. However, the observation that oligodendrocytes are able to wrap myelin around cylindrically shaped artificial fibers only coated with Poly-D-Lysine show that unspecific adhesion is sufficient for the formation of multi-lamellar myelin^12–14^. The three dimensional structure of myelin generated around artificial fibers in the absence of specific adhesive contacts—particularly at their ends and at the leading edge—remains to be determined. Notably, in the absence of axo-glial adhesion molecules the machinery necessary to generate myelin membrane remains intact. This is consistent with our results showing that the deletion of axo-glial adhesion molecules does not inhibit the production of myelin *per se*, but rather the coordinated guidance of the leading edge around the axon. Nevertheless, our data also indicate that in the absence of these adhesive systems mutant zebrafish oligodendrocytes generate shorter and fewer myelin sheaths per oligodendrocytes, possibly the dysfunctional leading edge results in a traffic jam and feed-back inhibition of myelin biogenesis. Even if most myelin sheaths appeared with abnormal structure in double mutants, some normal sheaths were generated. One reason why some internodes remain intact could be the slow growth of myelin in double mutants. Whether particular subclasses of oligodendrocytes^46,47^ or axons^48,49^ are more susceptible to undergo aberrant myelination remains to be determined. Interestingly, our data also show that the function of these adhesion molecules differs between the peripheral and central nervous system.

In this study, we combined data from two complementary experimental systems, as the basic mechanisms of myelination appear to be conserved between fish and mice^50^. Our trans-species approach allowed us to take advantage of the amenability for high-resolution live imaging in zebrafish and of the ultrastructural preservation of myelin structure for electron microscopy analyses in mice. The function of the two adhesive systems appears to be very well conserved between both systems with one exception. Depletion of paranodal adhesion molecules resulted in aberrant targeting of myelin membrane to neuronal cell bodies in zebrafish, but extensive cell body wrappings was not seen in mice. One possible explanation is that non-conserved inhibitory cues, such as neuronal junction adhesion molecule 2 or other inhibitory molecules, prevent cell body wrappings in mice^51^. Interestingly, we find that cell body wrappings were transiently observed in wild-type zebrafish at early stages of myelination. Thus, cell body wrapping is possibly a result of aberrant myelin generation that occurs during normal development and requires pruning or retractions, a process that may differ between mice and zebrafish. Notably, cell body wrappings were reduced in *caspr*^*−/−*^*mag*^*−/−*^ as compared to the single *caspr*^*−/−*^ mutant. Since excess myelin is inappropriately targeted to cell bodies ^52^, the reduction of myelin generation in *caspr*^*−/−*^*mag*^*−/−*^ may explain the relative decrease in cell body wrapping. A competitive balance between cell body wrappings and double myelination is another explanation.

Collectively, our study provides a mechanistic framework to explain how adhesion molecules act in the generation of myelin as a multilayered structure. This model not only fills a gap in our understanding of myelin biogenesis, but envisions that this knowledge will be valuable for our understanding of remyelination and myelin pathology in various neurological diseases in which myelin structure is perturbed.

## Methods

### Contact for reagents and resource sharing

Requests for further information, resources, and reagents should be directed to and will be fulfilled by Mikael Simons (msimons@gwdg.de).

### Zebrafish husbandry

All zebrafish procedures have been carried out with approval and according to the regulations of the District Government of Upper Bavaria (project license AZ55.2-1-54-2532-157). We used the existing transgenic zebrafish lines Tg(mbp:EGFP-CAAX) and Tg(Sox10:mRFP)^39,53^. The following zebrafish knockout lines have been generated by CRISPR/Cas9-mediated gene editing^54^ of Tg(mbp:EGFP-CAAX) fish: *cntn1b*^*−/−*^, *caspr*^*−/−*^, *nfascb*^*−/−*^ and *mag*^*−/−*^. Double mutants *cntn1b*^*−/−*^*mag*^*−/−*^ and *caspr*^*−/−*^*mag*^*−/−*^ were generated by crossbreeding of the F1 generation. The *mag*^*−/−*^ *del2/ins3* and the *mag*^*−/−*^ *del18/ins7* line were used to create *cntn1b*^*−/−*^*mag*^*−/−*^ and *caspr*^*−/−*^*mag*^*−/−*^, respectively. Zebrafish were housed at the DZNE fish facility in Munich according to local animal welfare regulations. Embryos were obtained by natural spawning and raised at 28.5 °C in E3 medium.

### Mouse husbandry

All mouse procedures have been carried out with approval and according to the regulations of the state government of Lower Saxony (project license AZ: 14/1729) and the District Government of Upper Bavaria (project license AZ55.2-1-54-2532-157). *Caspr*^*−/−*^*/Caspr2*^*−/−*^ (NMRI background) were generated by crossing *Caspr*^32^ and *Caspr2*^43^ mice. *Mag*^*−/−*^ (C57/B6J background) and *Cntn1*^*m1J−/−*^ were obtained from Jackson Laboratories, and grown on a mixed background of BALB/cByJ and C57/B6J)^55^. The following double and triple mutants were generated by crossbreeding: *Mag*^*−/−*^*Caspr*^*−/−*^, *Mag*^*−/−*^*Caspr2*^*−/−*^, *Mag*^*−/−*^*Caspr*^*−/−*^*Caspr2*^*−/−*^, *Mag*^*−/−*^*Cntn1*^*−/−*^. *Caspr*^*−/−*^*and Caspr2*^*−/−*^ single mutant mice were obtained by outbreeding against C57/B6J wild-type mice. Mice were housed at the MPI of Experimental Medicine in Göttingen (*Mag*^*−/−*^, *Caspr*^*−/−*^*/Caspr2*^*−/−*^), the DZNE in München (*Mag*^*−/−*^, *Caspr*^*−/−*^*/Caspr2*^*−/−*^) and the Institute of Neuronal Cell Biology, TU München (*Mag*^*−/−*^, *Cntn1*^*−/−*^).

Genotyping was done in a standard PCR reaction with 1 µl of extracted DNA and the following primers: *Caspr* wt: 5’-GAGAGGGAAGGGTGGATAAGGAC-3’ and 5’-ATTGCGGAGCGCTGGGGAGAGG-3’, *Caspr*^*−/−*^*:* 5’-ATTTCCCAACGGCAGGTT-3’ and 5’-TCGCCTTCTTGACGAGTTC-3’, *Caspr2*^*−/−*^: 5’-TCAGAGTTGATACCCGAGCGCC-3’, 5’-TGCTGCTGCCAGCCCAGGAACTGG-3‘ and 5’-TTGGGTGGAGAGGCTATTCGGCTATG-3‘, *Cntn1*^*−/−*^*:* 5’-TAGACCCATGCAAGCAGACA-3‘ and 5’-CAGGGCCCAAGTACCCTTAC-3‘, *Mag*^*−/−*^: 5’-TTGGCGGCGAATGGGCTGAC-3‘ 5’-CGGCAGGGAATGGAGACAC-3‘ and 5’-ACCCTGCCGCTGTTTTGGAT-3‘. To genotype the point mutation in *Cntn1*^*−/−*^ mice, PCR products were digested with BslI for 1 h at 55 °C. *Cntn1*^*−/−*^ mice were identified by fully undigested bands.

### Transgenic constructs

pBH-UAS:EGFP-Cntn1b was built as an analogous plasmid to pBH-UAS:EGFP-Cntn1a (David Lyons lab, Edinburgh) and generated by 4-fragment Gibson assembly. EGFP was positioned between the signal peptide and the coding sequence of Cntn1b (Ensembl: ENSDARG00000045685), in order to preserve the C-terminal GPI anchor. Cntn1b signal peptide and coding sequence were amplified from cDNA of 5 dpf AB wild-type zebrafish using the following primers: Cntn1b-SP: 5’-CTCACTTTGAGCTCCTCCACACGGCCAAGTACCATGGCGACCCCAG-3’ (forward) and 5’-AACAGCTCCTCGCCCTTGCTCACCATGGTTGCAGCTGCTAATAGGAAGAAGCAGG-3’ (reverse), Cntn1b: 5’-GACGAGCTGTACAAGATTAAACCCAGGATCTTTGAACCCAG-3’ (forward) and 5’-ATCTTATCATGTCTGGATCATCATCGATGCCTACAGGCCTAAAGTGGTCCAGG-3’ (reverse). EGFP and the vector backbone were amplified from pBH-UAS:EGFP-Cntn1a with the following primers: EGFP: 5’-AGCTGCAACCATGGTGAGCAAGGGCGAGGA-3’ (forward) and 5’-GATCCTGGGTTTAATCTTGTACAGCTCGTCCATGCC-3’ (reverse), pBH-UAS backbone: 5’-GCATCGATGATGATCCAGACATGATAAGAT-3’ and 5’-ACTTGGCCGTGTGGAGGAGCTCAA-3’. Assembly of the fragments was done with the NEBuilder® HiFi DNA Assembly Cloning Kit (New England Biolabs, E5520S). We generated pTol2-UAS:caspr-YFP (Ensembl: ENSDARG00000074524) and pTol2-UAS:nfascb-EGFP (Ensembl: ENSDARG00000074524) using PCR amplification and cloning using the Tol2kit^56^. We generated pTol2-UAS:caspr-YFP by amplifying the caspr coding sequence (including signal peptide) from 5dpf AB wild-type cDNA using following primers: 5’-GGGGACAAGTTTGTACAAAAAAGCAGGCTATGGATATCAGAATTCTTGCCC-3’ (forward) and 5’-GGGGACCACTTTGTACAAGAAAGCTGGGTATTCATTGGGACTTTCCTCCT-3’ (reverse). Amplified DNA was cloned into a pDONR221 vector by gateway BP-reaction using Gateway BP Clonase II (Thermo Fisher Scientific). The pTol2-UAS:caspr-YFP construct was subsequently assembled from p5E-UAS, pME-caspr, p3E-YFPpA and pDestTol2CG by gateway LR-reaction using Gateway LR Clonase II plus (Thermo Fisher Scientific). We generated pTol2-UAS:nfascb-EGFP by amplifying the caspr coding sequence (including signal peptide) from 5dpf AB wild-type cDNA using following primers: 5’-GGGGACAAGTTTGTACAAAAAAGCAGGCTGCCACCATGAAGTGTTGGAGGATTC-3’ (forward) and 5’-GGGGACCACTTTGTACAAGAAAGCTGGGTAAGCAAAAGAGTAGATGGC-3’ (reverse). Amplified DNA was cloned into a pDONR221 vector by gateway BP-reaction using Gateway BP Clonase II (Thermo Fisher Scientific). The pTol2-UAS:nfascb-EGFP construct was subsequently assembled from p5E-UAS, pME-nfascb, p3E-EGFPpA, and pDestTol2CG using Gibson assembly. Furthermore, we used mbp:mCherry-CAAX and sox10:Lifeact-tagRFP-t^40^.

### Microinjection and generation of mutant zebrafish

Transient expression of plasmids in zebrafish was done by injecting a 1:1 solution of plasmid DNA (25 ng/μl) and transposase mRNA (25–200 ng/μl) into fertilized eggs at one-cell stage. Embryos for imaging were treated with PTU from 8–24 hpf onwards to prevent pigmentation. Mutant zebrafish were generated by CRISPR/Cas9. Up to 3 guide RNAs against 1–2 exons per target were designed based on the following criteria: 1. prediction of high on-target activity, 2. absence of off-targets in genes, and 3. presence of a restriction site in close proximity to the Cas9 cleavage site that is unique within at least 150 bp upstream and downstream of the target sequence. gRNA predictions were made by free online tools (crispr.mit.eu from the Zhang lab, MIT, and CHOPCHOP, chopchop.cbu.uib.no)^57^. Target-specific CRISPR RNAs (crRNAs) were annealed with trans-activating CRISPR RNA (tracrRNA, all from Integrated DNA Technologies). We injected a 1:1-solution of complexed crRNA:tracrRNA oligos (1 mM) and Cas9 Protein (1.25 mg/ml, PNA Bio) into fertilized eggs at one-cell stage. Injected eggs were analyzed 3–5 days post-fertilization for genome modification of the target locus using restriction fragment length polymorphism of the PCR products. If positive, siblings from the same egglay were raised to adulthood and crossed with wild-type AB or Tg(mbp:gfp-CAAX) fish to create germline mutants with defined mutations in the *F*1 progeny. Frame-shift mutations in F1 fish were confirmed by Sanger sequencing and fish with identical mutations were crossed to generate *F*2 homozygous mutants. Gene inactivation was confirmed using quantitative real-time PCR of the respective gene products.

For genotyping, anesthetized zebrafish larvae or fin clips were lysed in Tris-EDTA buffer (TE) (pH = 8.0) and 1.7 mg/ml proteinase K for 4-8 h at 55 °C, following heat inactivation of Proteinase K^54^. 1–2 µl of lysate were used for PCR with the following primers: *caspr*^*−/−*^*:* 5’-CAAATACATGGTGCTGTACG-3’ and 5’-CCAACATTGTAAGCATAGACC-3’, *cntn1b*^*−/−*^: 5’-CGTCTTTAAATTTTACCTTAAGTGCC-3’ and 5’-TGCACTTTAACACAGATTAATGGAA-3’, *nfascb*^*−/−*^*:* 5’-AGAAGGCGGGGCTTAATATAAC-3’ and 5’-ATAAATGCAGTCTTGGTGAGCA-3’, *mag*^*−/−*^: 5’-CTCTTTCTCTAAACAGATGCAAGC-3’ and 5’-CGACAGAATTTTCATTGCTGG-3’. We checked for restriction fragment length polymorphism by incubating PCR products for 1 h at 37 °C with the following enzymes: *caspr*^*−/−*^: NcoI, *cntn1b*^*−/−*^: AciI, *nfascb*^*−/−*^: AciI, *mag*^*−/−*^: HindIII).

### RNA isolation and gene expression analysis

Brains from adult zebrafish (*cntn1b*^*−/−*^, *caspr*^*−/−*^, *mag*^*−/−*^) and 5dpf zebrafish larvae (*nfascb*^*−/−*^) were snap-frozen and stored at −80 °C. For RNA isolation, samples were homogenized in RLT Buffer Plus (Qiagen) for 30 min on ice, followed by centrifugation in QIAshredder columns (Qiagen, 79654) for 2 min at maximum speed. RNA was isolated from lysates of whole brains or single larvae with the RNeasy Mini kit (Qiagen, 74104) and retrotranscribed with the SuperScript III First-Strand Synthesis system (ThermoFisher, 18080051). Quantitative real-time PCRs were performed with PowerUp SYBR Green Master Mix (ThermoFisher, A25742), using an Applied Biosystems 7500 Fast Real-Time PCR system. The following primers were used: *cntn1b*: 5’-TGGAAGAAATCGGCGACACA-3’ and 5’-TTCAGAAACGCAGGAGTGGT-3’, *caspr*: 5’-ATGGCAGACGGTTTTCCTCA-3’ and 5’-ACCTCCCACCTCCATGACTC-3’, *nfascb*: 5’-TAGACATCGTGACGCAGGGA-3’ and 5’-TATCCTCTCAAGAGACCTGTAATCA-3’, *mag*: 5’-TAGAGGAAGGCACGGGAGAC-3’ and 5’-GGGGCAGAGGGAATGATTGG-3’, Elf1a 5’-AGCAGCAGCTGAGGAGTGAT-3’ and 5’-GTGGTGGACTTTCCGGAGT-3’. Relative quantification of gene expression was performed by the ΔΔCt method.

### Imaging in zebrafish

Zebrafish larvae at 3 to 11 dpf were anesthetized with tricaine, and mounted laterally in 0.8–1% low melting point agarose (ThermoFisher) on a glass bottom dish (#1.5 cover glass, IBL). After imaging, larvae were sacrificed and genotyped, if progeny from heterozygous incrosses was used. Fish were imaged at a Leica TCS SP8 confocal laser scanning microscope with automated moving stage and climate chamber (28.5 °C), using a 1.1 NA 40× water immersion objective and 488, 514 and 552 nm lasers. Single images (1248x1248 pixels) for quantification of myelinated cell bodies, myelinated area, number of myelinated sheaths per oligodendrocyte and myelin sheath length were acquired using a hybrid detector in counting mode, line accumulation of 4, and the pinhole at 0.8 airy units. 6 *z*-stack tiles (*z*-step = 0.33 µm) along the upper spinal cord were acquired and stitched using the LAS X (v.3.5) software. For caspr-YFP localization experiments, all caspr-YFP-positive sheaths within a hemi-spinal cord were imaged. Live imaging (1248 × 1248 pixels) *z*-stacks over time for quantification of extension and retraction events were acquired with a 40× water immersion objective (1.1 NA, using Zeiss Immersol W as immersion medium), a hybrid detector in photon counting mode, with 8× line accumulation, and a pinhole at 1.2 airy units. 13 *z*-stack tiles (*z*-step = 1 µm) along the spinal cord, starting at the neck were taken every 30 min. Live imaging of caspr-YFP localization was performed on selected caspr-YFP-positive sheaths, with time intervals of 30 min. A bright field channel was acquired simultaneously to surveil the health status of the animals, capturing the transmitted laser light with a TL-photomultiplier. Images were stitched using the LAS X (v3.5) software. All images of zebrafish show lateral views of the spinal cord with anterior to the left and dorsal to the top.

### Image analysis

Myelinated area in Tg(EGFP-CAAX) zebrafish was determined in Fiji as the total area of mbp:EGFP-CAAX-positive pixels from ROIs of manually thresholded maximum intensity projections. Myelinated cell bodies, sheath lengths, sheaths per oligodendrocytes and number of oligodendrocytes in zebrafish were quantified using the 3D surpass view of IMARIS (Bitplane). Sheath lengths were measured from sheaths in the dorsal spinal cord, including sheaths of commissural neurons that could be followed along their entire length. Fluorescence intensity profiles were analyzed in Fiji and quantifications were done in Excel. All images showing colocalizations (Figs. 2d, 3b, 7a, d, S1 and S3D) were deconvolved using the Huygens Essential software (Scientific Volume Imaging). In Supplementary Fig. 1, the background was removed using the Background Subtraction plugin of Fiji with a rolling ball radius of 80 pixels, prior to maximum intensity projection. Quantification of sheath retractions from ensheathed/myelinated cell bodies and axons in time lapse acquisitions was done on maximum intensity projections in Fiji and confirmed in *z*-stacks. Sheaths lengths of extending and retracting sheaths were measured per frame, plotted in Excel, and elongation and retraction rates were calculated from the slope of those consecutive data points that represented the main elongation/retraction event. Time-lapse movies were corrected for 3D drift in Fiji before maximum intensity projection. F-actin in fish was quantified as the area of LifeAct-RFP positive pixels normalized by the area of the myelin sheath in manually thresholded maximum intensity projections.

### Immunohistochemistry of mouse tissue

Mice were anesthetized with xylazin/ketamine or isoflurane and perfused with 2% or 4% PFA in PBS. Spinal cord, optic nerve and brain were removed and fixed in 2% or 4% PFA in PBS overnight. For cryosections, samples were transferred to 30% sucrose solution, embedded in optimal cutting temperature compound (OCT) after at least two days and stored at −80 °C. Longitudinal optic nerve and spinal cord sections (30 µm) were cut by cryostat (Leica). Sections were stored free-floating in cryoprotective solution (25% ethylene glycol, 20% glycerol, in PBS). Sections were transferred to PBS in 24 well plates, permeabilized and blocked for 1 h with 0.2% triton-x-100, 2.5% Fetal Bovine Serum (FBS), 2.5% Bovine Serum Albumin and 2.5% fish gelatin in PBS. Primary antibodies were diluted in blocking solution containing triton-x-100 and applied overnight at 4 °C. We used rabbit anti-MBP (Dako, 1:1000), mouse anti-APC (merck clone CC1, 1:500), rabbit anti-Nav1.6 (Alomone, 1:200), ms IgG1 anti-Caspr (neuromab, 1:1000) and ms IgG2a anti-AnkG (neuromab, 1:250). Sections were washed 3 times with PBS before secondary antibodies (1:1000) were added and incubated for 1 h at room temperature. Sections were washed again, mounted to glass slides using Prolong Diamond antifade mountant and dried overnight. For vibratome sections, PFA-fixed brains were cut in sections of 150 µm using a vibratome (Leica). Sections were incubated in PBS-GT (0.2% gelatin, 1% triton-x-100) at 37 °C overnight. Staining was performed according to Belle et al.^58^. Antibodies were diluted in PBS-GT, added to slices and incubated for 3 days at 37 °C. Six PBS-GT washing steps (30 min) were applied and sections were incubated with secondary antibodies for another 2 days. Sections were again washed 6 times, mounted to glass slides using Prolong Diamond antifade mounting and dried overnight. Images were acquired using Leica SP5 and Leica SP8 confocal laser scanning microscopes.

### Fixation for electron microscopy

For EM of zebrafish tissue, 10dpf zebrafish larvae were fixed for TEM in 2% glutaraldehyde, 2% paraformaldehyde (EM grade, Science Services, Munich, Germany) and 2 mM CaCl_2_ in 0.1 M sodium cacodylate buffer at pH 7.4 using a BioWave (Pelco) microwave. In brief, fish were anesthetized in tricaine and heads were removed for genotyping. Body trunks were transferred into fixative on ice and microwave processed at multiple cycles at 100 W and 450 W. Fish were kept in fixative for at least 5 days. Mouse tissue for EM was either fixed by conventional chemical fixation or high pressure freezing. Mice were euthanized by cervical dislocation and decapitated at the age of 12 and 21 days postnatal. Mice at the age of 15-16 days postnatal were perfused with 4% PFA to allow tissue extraction for immunohistochemistry at the same time. For chemical fixation, optic nerves and sciatic nerves were removed immediately and transferred to Karlsson-Schulz-solution (4% PFA, 2.5% glutaraldehyde, 0.5% NaCl in 0.1 M phosphate buffer, pH = 7.3) for 1–4 days of fixation at 4 °C. Vertebral columns were removed en bloc and transferred to fixative, followed by dissection of spinal cords after two days and fixation for 2–3 additional days. For fixation by high pressure freezing, optic nerves were removed immediately and cryofixed by high pressure freezing in a HPM100 (Leica) within 5 min post decapitation, using polyvinylpyrrolidone as a filler. Samples were constantly kept under liquid nitrogen to prevent thawing and formation of ice crystals. Freeze substitution was performed in a Leica AFS II at −90 °C and samples were processed using the tannic acid-OsO_4_ protocol.

### Transmission electron microscopy (TEM)

After fixation for at least 5 days, fish were postfixed in 2% osmium tetroxide in 0.05 M imidazole and 0.1 M sodium cacodylate followed by further contrasting in 1% tannic acid, saturated uranyl acetate, dehydrated into 100% acetone and embedded in Epon resin (Serva). After ultrathin sectioning, grids (Leica UC7 ultramicrotome) were contrasted with 1% uranyl acetate and Ultrostain (Leica). Images were acquired using a JEOL JEM1400 plus TEM equipped with a Ruby 8Mpx CCD camera. Data analysis was carried out using Fiji. Mouse samples were postfixed in 1% OsO_4_, followed by en bloc uranyl acetate incubation, graded dehydration in ethanol and embedding in Epoxy resin. After ultrathin (50 nm) sectioning, grids (Leica UC7 ultramicrotome) were contrasted by 1% uranyl acetate and Ultrostain (Leica). Images from *Cntn1*^*−/−*^*Mag*^*−/−*^ animals and respective controls were acquired with a JEOL JEM1400 plus TEM equipped with a Ruby 8 Mpx CCD camera. Images from *Caspr*^*−/−*^*Caspr2*^*−/−*^*Mag*^*−/−*^ samples and respective controls were acquired with the LEO 912 Omega electron microscope (Zeiss) using an on-axis 2k CCD camera (TRS). Data analysis was carried out using Fiji and Adobe Photoshop. 10 randomly picked cross-section areas (225 µm^2^ each, ×7.000 magnification) per animal were used to count double myelinated axons and to quantify the ratio of myelinated vs. unmyelinated axons. Overgrown nodes in longitudinal sections were counted in the area of one grid hexagon (26.000 µm^2^) per animal. The area was imaged at ×3000 magnification and images were stitched together.

### Focused ion beam scanning electron microscopy (FIB-SEM)

*Caspr*^*−/−*^*Caspr2*^*−/−*^*Mag*^*−/−*^ samples were prepared by high pressure freezing and embedded in EPON and FIB-SEM was performed at a Zeiss Crossbeam 540 FIB-SEM. Images were acquired with a 3 nm pixel size in *x/y* and a *z*-depth of 50 nm. *Cntn1*^*−/−*^*Mag*^*−/−*^ embedded samples were trimmed with a razor blade and pieces were mounted with conductive silver colloid (Plano, Wetzlar, Germany) onto standard aluminum stubs (Plano, Wetzlar, Germany). Specimens were carbon coated (20 nm) by evaporation (Cressington Scientific Instruments UK, Waterford, UK). Samples were milled and imaged with an Auriga 40 FIB/SEM workstation operating under SmartSEM (Carl Zeiss Microscopy GmbH, Oberkochen, Germany) or Atlas-3D (Fibics ncorporated, Ottawa, Canada). Ion beam currents of 50 pA–10 nA were used. The milling rate was set to 2 nm slices, which allows the adjustment of the *z*-resolution in 2 nm steps at any time during the FIB/SEM run. SEM images were recorded with an aperture of 60 μm in the high current mode at 1.5 kV of the inlens EsB detector with the EsB grid set to −800–120 V. Voxel sizes of 5 × 5 × 20 nm were chosen. Images series of 800 consecutive sections were recorded. In the synchronous mode of the ATLAS-System, the milling current and depth were adjusted to match with exposure time of the SEM (1 min). Automatic correction of focus (auto tune) and astigmatism (auto stig) was applied every 30 min. FIB/SEM image stacks were aligned, segmented and 3D reconstructed in Amira (FEI Company).

### Automated tape-collection ultramicrotome SEM (ATUM-SEM)

Fixed mouse optic nerve samples were en bloc stained by a standard rOTO protocol^59^ in a sequence of reduced 2% osmium tetroxide in 1.5% potassium ferrocyanide in 0.1 M cacodylate buffer pH 7.4, 1% aqueous thiocarbohydrazide (TCH) and 2% aqueous osmium tetroxide including washing steps. After overnight incubation in 1% uranylacetate at 4 °C, samples were contrasted in 0.0665% lead aspartate, dehydrated and infiltrated with Epon. Blocks were trimmed by 200 µm to expose a rectangular tissue block using a TRIM90 knife (Diatome) on a Powertome ultramicrotome (RMC). Consecutive sections were taken with a diamond ultra knife (Diatome) at 150 nm thickness and collected on plasma-treated, carbon-coated Kapton tape (kindly provided by Richard Schalek, Jeff Lichtman, Harvard)^60^. Kapton strips with tissue sections were assembled onto carbon tape (Science Services), mounted onto a 4-inch silicon wafer (Siegert Wafer) and grounded with adhesive carbon tape strips. Section images were acquired on a Crossbeam Gemini 340 SEM (Zeiss) in backscatter mode at 8 kV (high gain) at 7.0 mm WD and 60 µm aperture. In ATLAS5 Array Tomography (Fibics, Ottawa, Canada) the entire wafer was imaged at 6000 nm/pixel followed by mapping and medium resolution (100 nm/pixel) imaging of individual tissue sections. A region of interest comprising 55 × 55  µm on 367 sections on two wafers was automatically acquired at 4 nm/pixel. Images were aligned using Fiji TrakEM2^61^. Image analysis was done in Fiji. For EM of zebrafish, embedded *cntn1b*^*−/−*^ (10 dpf) fish were cut on the ATUMtome (Powertome, RMC) in 200 nm sections. Five hundred semithin sections were collected onto carbon-coated Kapton tape, assembled on wafers (3 per fish) and manually screened for cell body myelination by SEM imaging.

### Statistics

Statistics were performed in R and GraphPad Prism. All samples were tested for normality and equal variances. One-way ANOVA was followed by pairwise student’s *t*-test with Bonferroni correction. If ANOVA was not applicable, pairwise Wilcoxon-signed-rank test or Kruskal-Wallis ANOVA was performed, using Bonferroni or Dunn’s correction. Data in the text is presented as mean ± s.d.

### Reporting summary

Further information on research design is available in the Nature Research Reporting Summary linked to this article.

## Supplementary information

Supplementary Information

Peer Review

Reporting Summary

Description of Additional Supplementary Files

Supplementary Movie 1

Supplementary Movie 2

Supplementary Movie 3

Supplementary Movie 4

Supplementary Movie 5

Supplementary Movie 6

Supplementary Movie 7

Source Data

## Data Availability

The authors declare that the data supporting the findings of this study are available within the paper and its supplementary information files. The source data underlying Figs. 1b, c, f, 2b, d, f-h, j, 3c, d, f, 4a–d, 5b,c, 6b, e–g, i, and Supplementary Figs. 1f–i, 2b, c, e, g, i, k–m, 3a, c–e, 4b, c, e, f, 5b–d, e, f, h, j, l, 6a, e are provided as a Source Data file.
