# Supplementary material for: Two adhesive systems cooperatively regulate axon ensheathment and myelin growth in the CNS
Source: Nat Commun. 2019 Oct 22;10:4794. doi: 10.1038/s41467-019-12789-z (PMC6805957; doi:10.1038/s41467-019-12789-z)
Supplement: Supplementary file 1 — Supplementary Information [file 41467_2019_12789_MOESM1_ESM.pdf]

Supplementary Information

Two adhesive systems cooperatively regulate axon ensheathment and myelin growth in the CNS

by Djannatian and Timmler et al.

Supplementary Figure 1 – Cntn1b, Caspr and Nfascb localize to paranodes in zebrafish

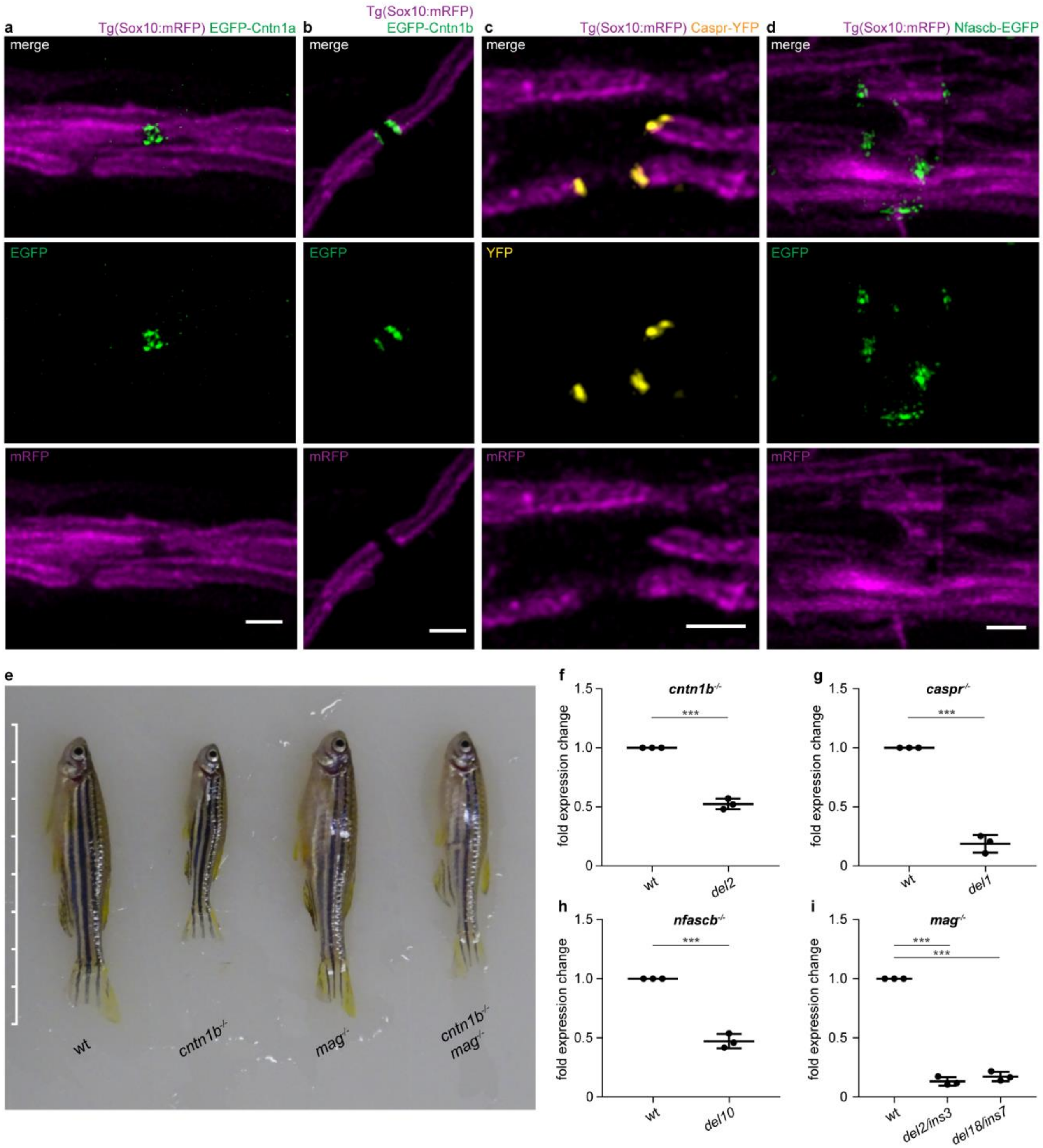

a-d Co-expression of UAS-driven fusion proteins with HuC:Gal4 (for neuronal expression) or Sox10:Gal4 (for glial expression) reveals nodal expression of EGFP-Cntn1a (**a**, 4 dpf) and paranodal expression of EGFP-Cntn1b (**b**, 7 dpf), Caspr-YFP (**c**, 5 dpf) and Nfascb-EGFP (**d**, 3

dpf) in Tg(Sox10:mRFP) dorsal spinal cord axons. **e** Phenotypes of adult wild-type (wt) and mutant zebrafish (90 dpf). Scale bar ticks represent 0.5 cm. **f-i** Validation of zebrafish mutant lines by quantitative PCR using the ΔΔCt method. Graphs represent fold expression change of mutant fish compared to age-matched wt fish. *cntn1b*$^{-/-}$ *del2* (**f**, *n* = 3 fish, unpaired two sided t test), *caspr*$^{-/-}$ *del1* (**g**, *n* = 3, unpaired two sided t test), *nfascb*$^{-/-}$ *del10* (**h**, *n* = 3, unpaired two sided t test), *mag*$^{-/-}$ *del2/ins3* and *mag*$^{-/-}$ *del18/ins7* (**i** , *n* = 3, one-way ANOVA, $p < 0.0001$). Bonferroni-corrected p values: ***p < 0.001. Data are presented as means ± s.d. Scale bars, 2 μm. Source data are provided as a Source Data file.

Supplementary Figure 2 – Mag and paranodal adhesion molecules regulate myelin growth, but do not change oligodendrocyte number in zebrafish

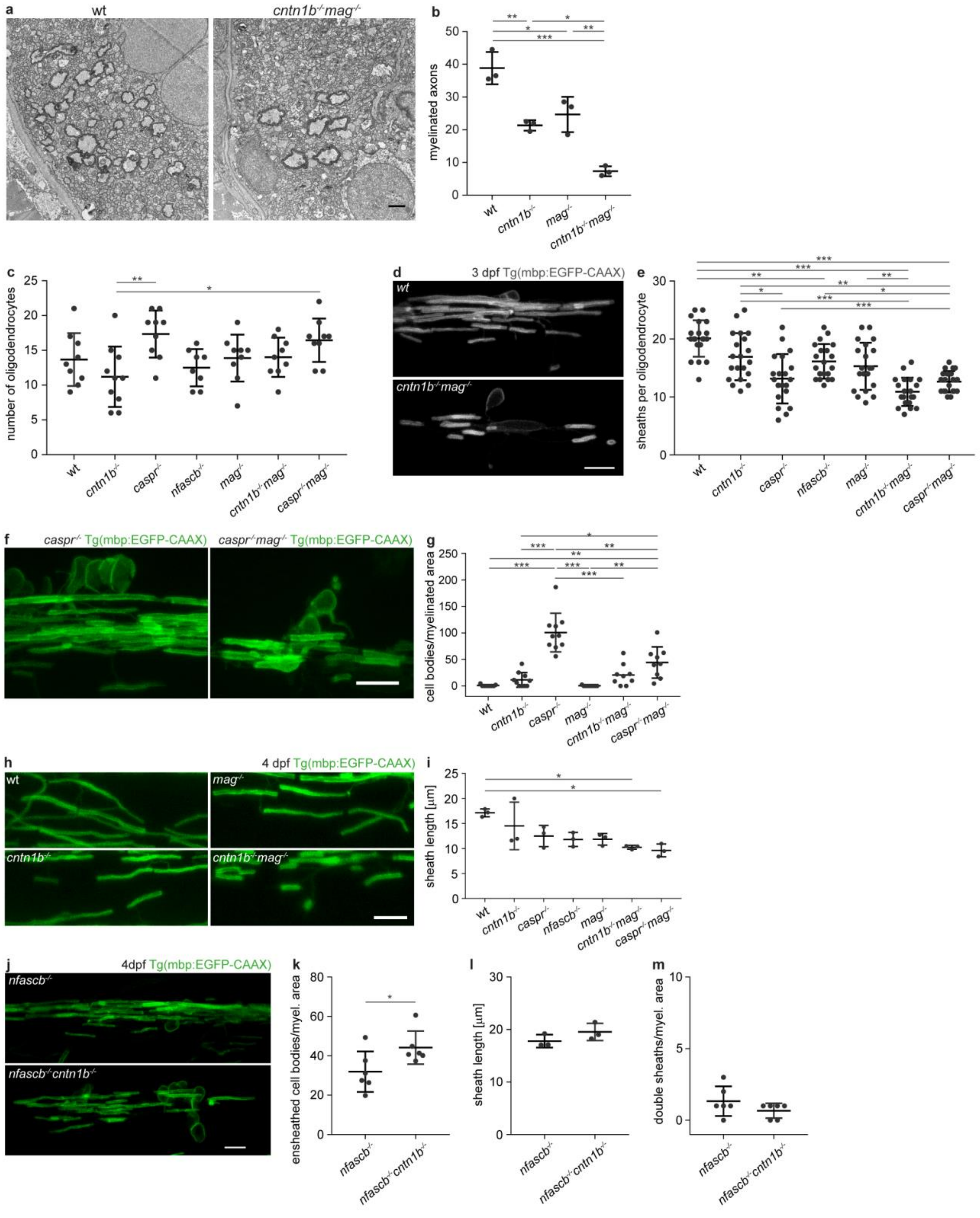

a Electron micrographs reperesent dorsal spinal cord cross-sections of 10 dpf wt and *cntn1b*$^{-/-}$ *mag*$^{-/-}$zebrafish. **b** Total numbers of myelinated axons in electron micrographs of 10 dpf wt and mutant dorsal spinal cord cross-sections. $n = 3$ fish, one-way ANOVA: $p < 0.0001$. **c** Number of oligodendrocytes in 3 dpf anterior dorsal spinal cords of wt and mutant Tg(mbp:EGFP-CAAX) zebrafish larvae. n = 8-10 fish, one-way ANOVA: $p < 0.0049$. **d** Myelin sheaths of single oligodendrocytes in 3 dpf wild-type (top) and *cntn1b*$^{-/-}$*mag*$^{-/-}$ (bottom) fish. **e** Number of myelin sheaths per oligodendrocyte in 3 dpf wt and mutant fish. Sheaths from 20 oligodendrocytes per genotype from 5-7 fish were quantified. One-way ANOVA: $p < 0.0001$. **f** Ensheathed cell bodies in 4 dpf *caspr*$^{-/-}$ (reproduced from Fig. **1a**) and *caspr*$^{-/-}$*mag*$^{-/-}$ fish. **g** Ensheathed cell bodies per myelinated area at 4 dpf (n = 8-10, one-way ANOVA: $p = 0.0001$). Quantifications for wt and single mutants were reproduced from Fig. **1b**. **h** Representative wild type (wt) and mutant myelin sheaths of zebrafish commissural neurons at 4 dpf. **i** Sheath length at 4 dpf (means of 30 sheaths per animal, $n = 3$, one-way ANOVA, $p < 0.0001$). **j-m** CNS myelination of 4 dpf *nfascb*$^{-/-}$*cntn1b*$^{-/-}$ fish in comparison to *nfascb*$^{-/-}$littermates. (**j**) Representative images of CNS myelin. (**k**) Ensheathed cell bodies per myelinated area ($n = 6$, unpaired two-sided t test: $p = 0.0482$). (**l**) Sheath length (means of 30 sheaths per animal, $n = 3$, unpaired two-sided t test, $p < 0.2111$). (**m**) Double sheaths (sheaths with fluorescence intensity steps combined with caliber changes, as depicted in Fig. **4a**) normalized to myelinated area ($n = 6$, unpaired two-sided t test, $p < 0.1877$). Confocal images (**d,f,h,j**) are maximum intensity projections of Tg(mbp:EGFP-CAAX) zebrafish dorsal spinal cord. Bonferroni-corrected p values, *0.05, **<0.01, ***<0.001. Data are presented as means ± s.d. Scale bars, 1 µm (**a**), 10 µm (**d,f,h,j**). Source data are provided as a Source Data file.

Supplementary Fig. 3 – Double myelin sheaths and sheath retractions in zebrafish

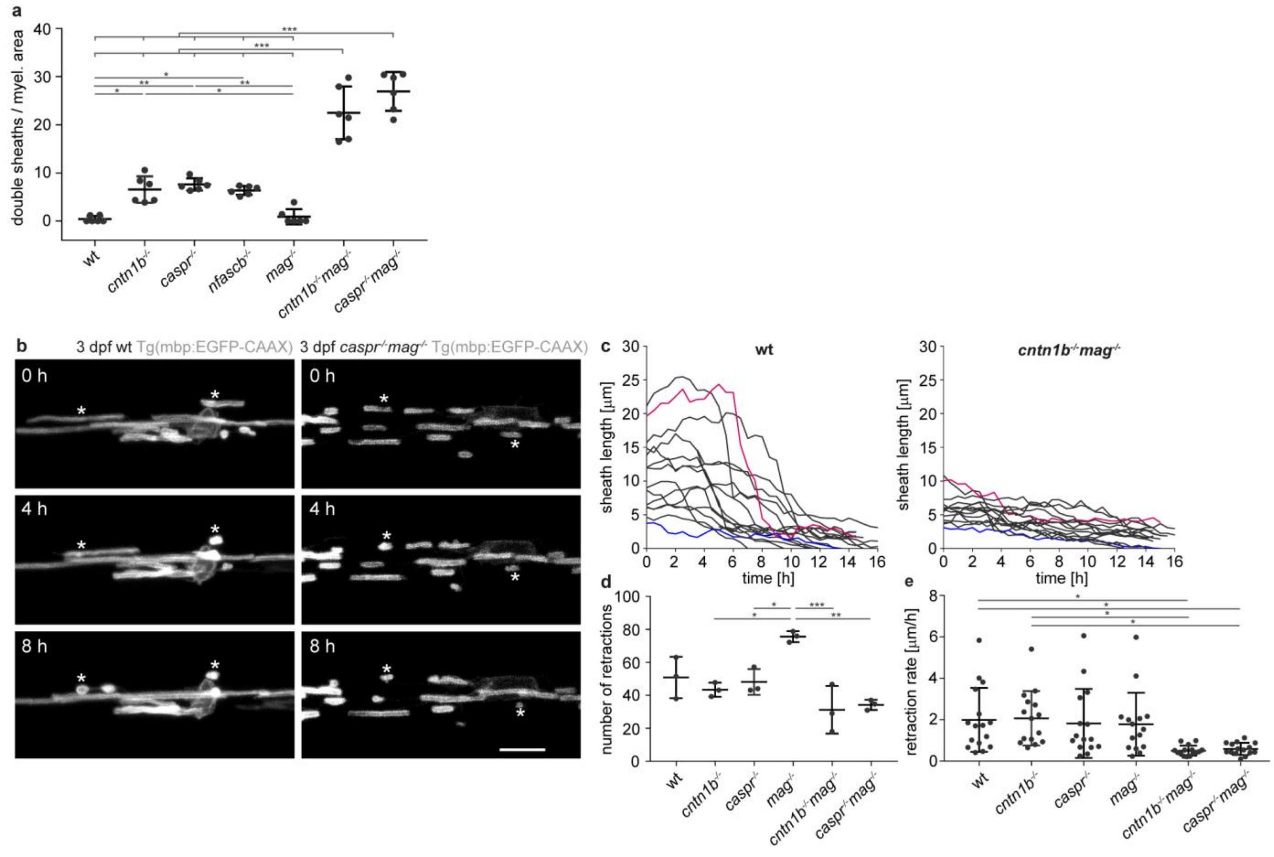

a Quantification of double myelin sheaths (sheaths with fluorescence intensity steps combined with caliber changes, as depicted in Fig. **4a**) normalized to myelinated area at 10 dpf (n = 6 fish per genotype, one-way ANOVA: $p < 0.0001$. **b-e** Myelin sheath retractions at 3 dpf (30 min interval, 15-16 h time lapse experiments). Asterisks in (**b**) represent retracting sheaths. Sheath length over time is shown in (**c**) (15 sheaths from 3 animals). The fastest (magenta) and slowest (blue) retracting sheath are highlighted. Total number of retractions during the time lapse experiment is shown in (**d**, n = 3 fish). One-way ANOVA: $p < 0.0007$. Retraction rates (**e**) were calculated from (**c**) analogous to Fig. **2g** (n = 15-16 sheaths from 3 fish). One-way ANOVA: $p <$ 0.0005. Confocal images (**b**) are maximum intensity projections of Tg(mbp:EGFP-CAAX) zebrafish dorsal spinal cord. Bonferroni-corrected p values, *0.05, **<0.01, ***<0.001. Data are presented as means ± s.d. Scale bar, 10 µm. Source data are provided as a Source Data file.

Supplementary Fig. 4 – Phenotypes of Mag and paranodal adhesion molecule deficient mice

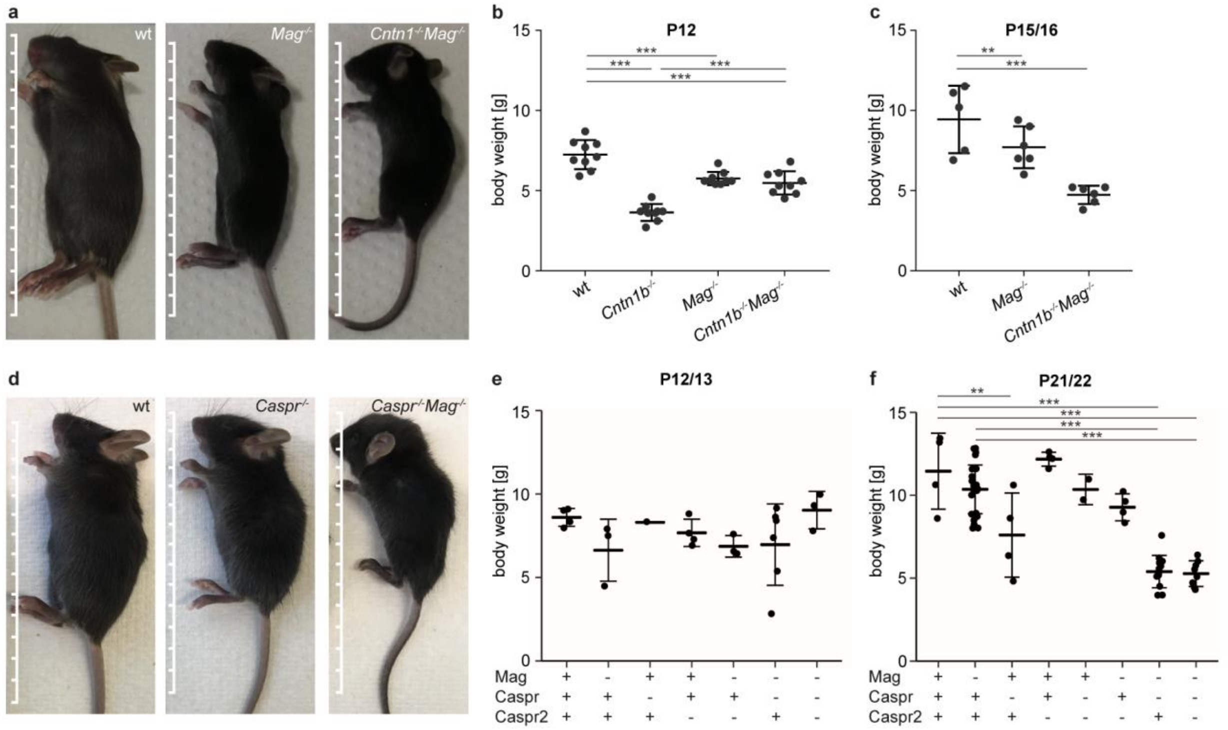

a Anesthetized wt, *Mag*$^{-/-}$ and *Cntn1*$^{-/-}$*Mag*$^{-/-}$ mice at P15/16. **b** Body weights of wt and mutant mice at P12 (*n* = 9 mice). One-way ANOVA: $p < 0.0001$. **c** Body weights of wild-type and mutant mice at P15/P16. *Cntn1*$^{-/-}$ mice reach a humane endpoint around P12 and are therefore not represented in the analysis. *n* = 5 (wt), n = 6 (*Mag*$^{-/-}$ and *Cntn1*$^{-/-}$*Mag*$^{-/-}$). One-way ANOVA: $p <$ 0.0001. **d** Anesthetized wt, *Caspr*$^{-/-}$ and *Caspr*$^{-/-}$*Mag*$^{-/-}$ mice at P21/22. **e** Body weights of wt and mutant mice at P12/13. *n* = 6 (*Caspr*$^{-/-}$*Mag*$^{-/-}$), *n* = 4 (wt, *Caspr2*$^{-/-}$), *n* = 3 (*Mag*$^{-/-}$, *Caspr2*$^{-/-}$*Mag*$^{-/-}$, *Caspr*$^{-/-}$*Caspr2*$^{-/-}$*Mag*$^{-/-}$), *n* = 1 (*Caspr*$^{-/-}$). One-way ANOVA: $p = 0.3735$. **f** Body weights of wild-type and mutant mice at P21/22. *n* = 25 (*Mag*$^{-/-}$), *n* = 8 (*Caspr*$^{-/-}$*Caspr2*$^{-/-}$*Mag*$^{-/-}$), *n* = 4 (wt, *Caspr*$^{-/-}$, *Caspr2*$^{-/-}$, *Caspr2*$^{-/-}$*Mag*$^{-/-}$), *n* = 13 (*Caspr*$^{-/-}$*Mag*$^{-/-}$), *n* = 2 (*Caspr*$^{-/-}$*Caspr2*$^{-/-}$). One-way ANOVA: $p < 0.0001$. Scale bar ticks represent 0.5 cm (**a, d**). Bonferroni-corrected *p* values: **<0.01, ***<0.001. Data are presented as means ± s.d. Source data are provided as a Source Data file.

Supplementary Figure 5 – Hypomyelination in Mag/paranodal adhesion molecule-deficient mice

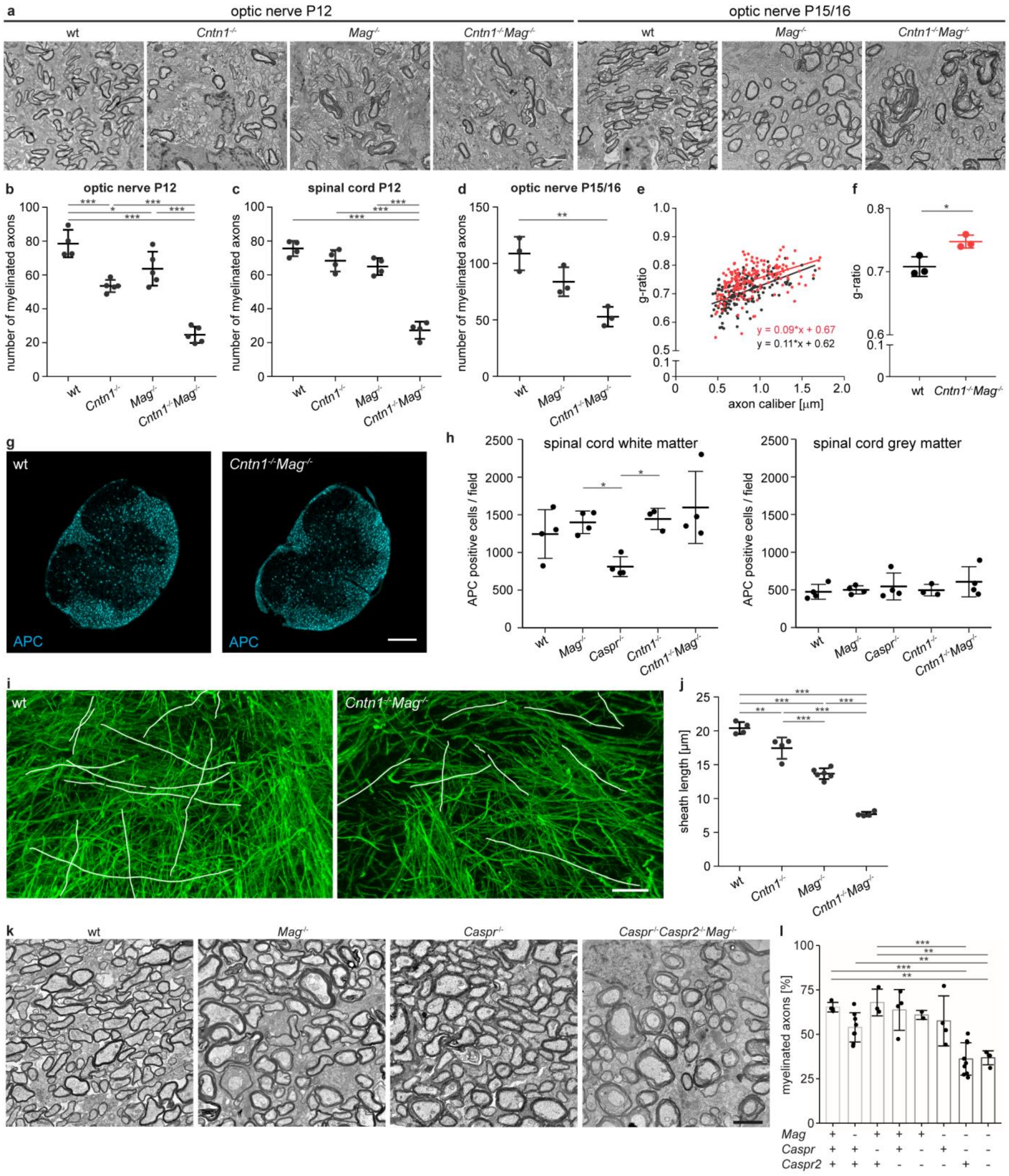

a Representative electron micrographs of optic nerve cross sections of P12 and P15/16 wild-type (wt) and mutant mice. **b** Number of myelinated axons in optic nerve cross sections of P12 wt and mutant mice. *n* = 4 (wt), *n* = 5 mice (C*ntn1*$^{-/-}$, *Mag*$^{-/-}$ and C*ntn1*$^{-/-}$*Mag*$^{-/-}$). One-way ANOVA: $p < 0.0001$. **c** Number of myelinated axons in thoracic spinal cord cross sections of P12 wt and mutant animals (n = 4 mice). One-way ANOVA: $p < 0.0001$. **d** Number of myelinated axons in optic nerve cross sections of P15/16 wt and mutant mice (*n* = 3). One-way ANOVA: $p < 0.0045$. **e** Quantification of myelin thickness in P15/16 optic nerves by g-ratio analysis and axon caliber distribution for wt and *Cntn1*$^{-/-}$*Mag*$^{-/-}$ mice (*n* = 3). Only normal appearing myelinated axons were quantified. **f** g-ratios of P15/16 optic nerves in wt (black) and *Cntn1*$^{-/-}$*Mag*$^{-/-}$ (red) mice (*n* = 3). Unpaired two-tailed t-test. **g** Representative confocal images of wt and *Cntn1*$^{-/-}$*Mag*$^{-/-}$ spinal cord cross sections (P12), stained for APC (clone CC1). **h** Quantification of APC-positive cells per field of view (~1.8 x 1.8 mm) in grey matter (left) and white matter (right). *n* = 3 (*Cntn1*$^{-/-}$), *n* = 4 (all other genotypes). One-way ANOVA: $p = 0.0179$ (white matter), $p = 0.685$ (grey matter). **i** MBP staining of P12 vibratome brain sections (thalamus) of wt and *Cntn1*$^{-/-}$*Mag*$^{-/-}$ mice. White lines indicate representative sheaths followed along their entire length. **j** Quantification of individual sheath lengths in wt and mutant animals (*n* = 3). Pairwise Wilcoxon rank sum test. **k** Representative electron micrographs of optic nerve cross sections of P21/P22 wild-type (wt), *Caspr*$^{-/-}$, *Mag*$^{-/-}$ and *Caspr*$^{-/-}$*Caspr2*$^{-/-}$*Mag*$^{-/-}$ mice. **l** Myelinated axons in wt and mutant P21/22 mice, 10 frames analyzed (219 μm^2) per animal. *n* = 9 (*Caspr*$^{-/-}$*Mag*$^{-/-}$), *n* = 8 (*Mag*$^{-/-}$), *n* = 4 (wt, *Caspr2*$^{-/-}$, *Caspr2*$^{-/-}$*Mag*$^{-/-}$, *Caspr*$^{-/-}$*Caspr2*$^{-/-}$*Mag*$^{-/-}$), *n* = 3 (*Caspr*$^{-/-}$), *n* = 2 (*Caspr*$^{-/-}$*Caspr2*$^{-/-}$). One-way ANOVA: $p < 0.0001$. Bonferroni-corrected *p* values: *<0.05, **<0.01, ***<0.001. Data are presented as means ± s.d. Scale bars, 2 μm (**a, k**), 40 μm (**i**), 250 μm (**g**). Source data are provided as a Source Data file.

Supplementary Figure 6 – Cntn1$^{-/-}$ and MAG-/- mouse spinal cord lacks cell body wrappings

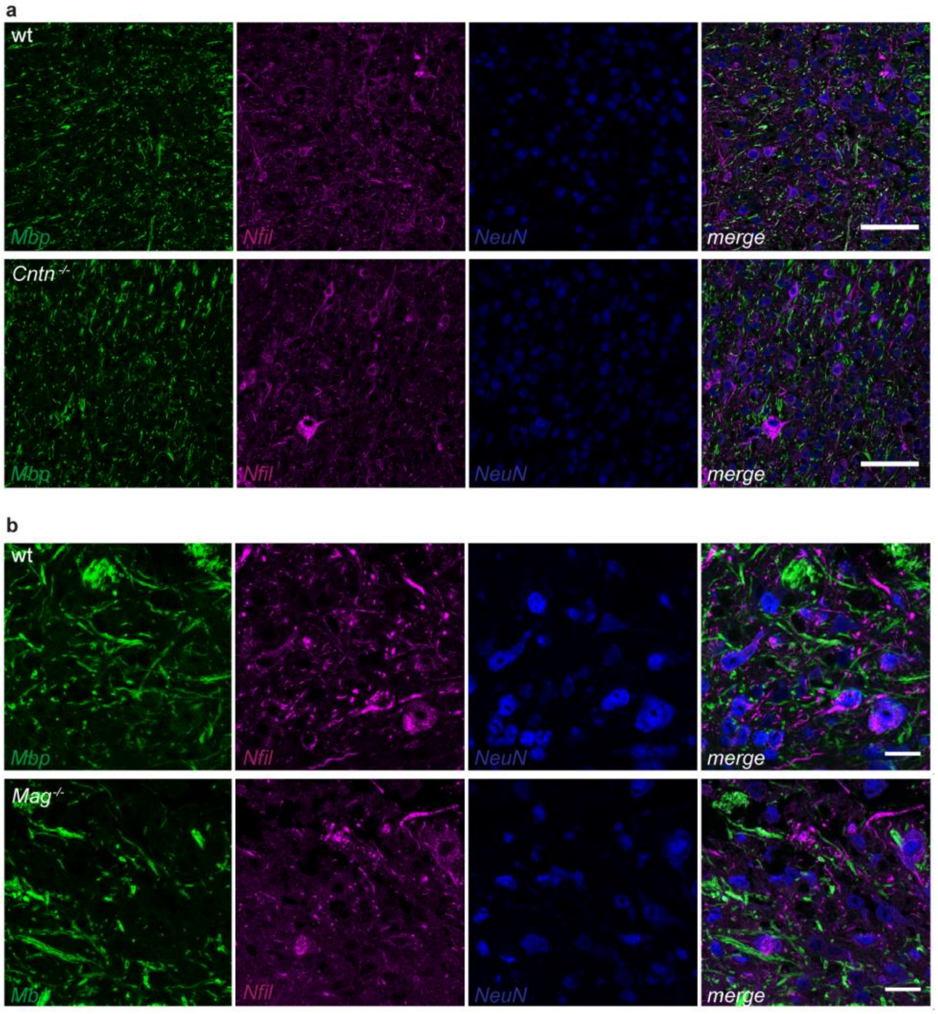

a, b Immunostainings of wildtype and *Cntn1*$^{-/-}$ (**a**) and MAG-/- (**b**) mouse spinal cord dorsal horn, labelling myelin (Mbp), axons (Nfil) and neuronal cell bodies (NeuN). Cell body ensheathments were not found. Scale bars, 100 µm (**a**) and 20 µm (**b**).

Supplementary Figure 7 – Double myelinated axons in Mag/paranodal adhesion molecule deficient mice in CNS, but not PNS

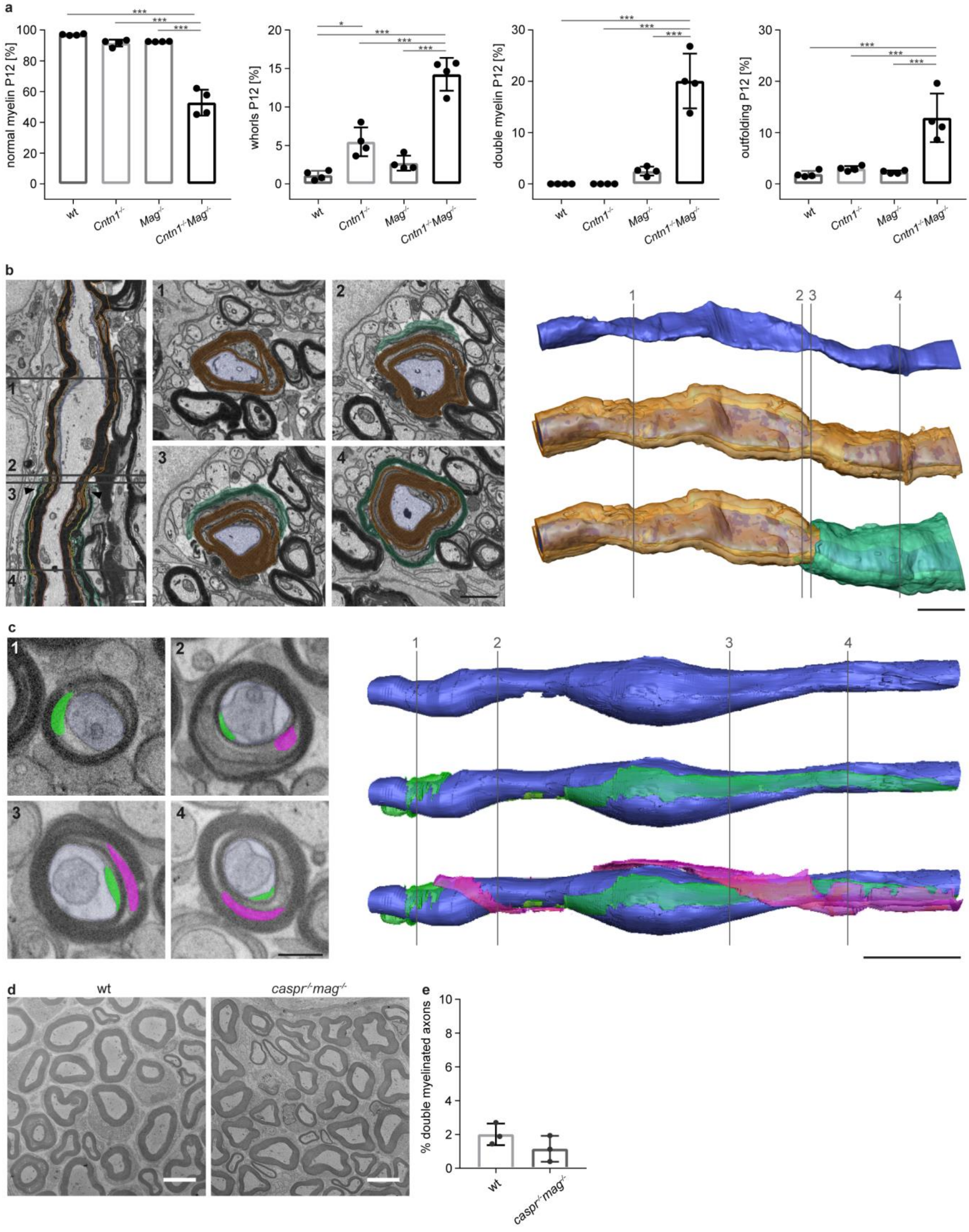

a Quantification of normal myelinated axons and myelin pathologies in P12 optic nerve cross sections (n = 4 mice). One-way ANOVA: $p < 0.001$ in each category. **b** Serial block-face SEM (FIB-SEM) of a P15 *Cntn1*$^{-/-}$*Mag*$^{-/-}$ optic nerve (22x15x18 µm volume). Longitudinal section (left) shows a myelinated axon (axon, blue, and compact myelin, orange) surrounded by a double layer of myelin (green) in the bottom part of the image. Arrows indicate paranodal loops on myelin, which identify the double layer as an independent myelin segment. Cross-sections are depicted at different *z*-levels. 3D reconstruction (right) shows the axon alone (top row), compacted myelin around the axon (middle row) and double myelin covering part of the myelinated axon (bottom row). Numbering in the longitudinal section and 3D reconstruction refers to cross-sections. **c** Serial block-face SEM (FIB-SEM) of a P21 *Caspr*$^{-/-}$*Caspr2*$^{-/-}$*Mag*$^{-/-}$ optic nerve (13 µm x 6 µm x 35 µm). Cross-sections (left) at different *z*-levels show the localization of two leading edges within one myelin sheath: one leading edge (green) is attached to the axon (blue), while the other leading edge (magenta) is found in between non-compacted layers. 3D reconstruction (right) shows the axon alone (top row), the leading edge that stays attached to the axon (middle row) and, in addition, the leading edge that is localized in between previously formed myelin layers (bottom row). Numbering refers to cross-sections. **d** Representative electron micrographs of sciatic nerve cross sections of P21 wt and *Caspr*$^{-/-}$*Mag*$^{-/-}$ mice. **e** Quantification of double myelinated axons in sciatic nerve cross sections (n = 3, unpaired two-sided t test, $p < 0.2119$). Bonferroni-corrected p values: *<0.05, **<0.01, ***<0.001. Data are presented as means ± s.d. Scale bars, 500 nm (**b** (longitudinal section), **c** (cross-sections)), 1 µm (**b** (cross-sections)), 2 µm (**b** and **c**, 3D reconstructions), 5 µm (**d**). Source data are provided as a Source Data file.
