# Supplementary material for: Two adhesive systems cooperatively regulate axon ensheathment and myelin growth in the CNS
Source: Nat Commun. 2019 Oct 22;10:4794. doi: 10.1038/s41467-019-12789-z (PMC6805957; doi:10.1038/s41467-019-12789-z)
Supplement: Supplementary file 4 — Description of Additional Supplementary Files [file 41467_2019_12789_MOESM4_ESM.docx]

**Description of Additional Supplementary Files**

File name: Supplementary Movie 1

Description: Time-lapse movie of myelin sheath retraction from a neuronal cell body. Myelin sheath retraction from a neuronal cell body in a 3 dpf wt Tg(mbp:EGFP-CAAX) zebrafish (selected frames shown in Fig. 1e). Images are maximum intensity projections of a 3D z-stack and show a representative detail of the anterior dorsal spinal cord.

File name: Supplementary Movie 2

Description: Time-lapse movie of myelin sheath elongation in wt and *cntn1b*^-/-^*mag*^-/-^ zebrafish. Elongation of myelin sheaths in 3 dpf wt and *cntn1b*^-/-^*mag*^-/-^Tg(mbp:EGFP-CAAX) zebrafish (selected frames shown in Fig. 2e). Images are maximum intensity projections of a 3D z-stack and show a representative detail of the anterior dorsal spinal cord.

File name: Supplementary Movie 3

Description: Time-lapse movie of myelin sheath retraction in wt and *caspr^-^/-mag-/-* zebrafish

Myelin sheath retractions in 3 dpf wt and *caspr*^-/-^*mag*^-/-^ Tg (mbp:EGFP-CAAX) zebrafish (selected frames shown in Supplementary Fig. 2h). Images are maximum intensity projections of a 3D z-stack and show a representative detail of the anterior dorsal spinal cord.

File name: Supplementary Movie 4

Description: 3D reconstruction from FIB-SEM of a P15 *Cntn1*^-/-^*Mag*^-/-^ double myelinated axon. 3D reconstruction of a double myelinated axon with one leading edge from a 22 µm x 15 µm x 18 µm FIB-SEM volume. A second myelin segment (green) is found on top of myelin (orange) on an axon (blue).

File name: Supplementary Movie 5

Description: FIB-SEM of a P15 *Cntn1*^-/-^*Mag*^-/-^ double myelinated axon with two leading edges. Cross-sections of a double myelinated axon followed through the z dimension of a 22 µm x 15 µm x 18 µm FIB-SEM volume. 3D reconstruction of a double myelinated axon with two leading edges from a 22 µm x 15 µm x 18 µm FIB-SEM volume. One leading edge (green) is in contact with the axon (blue) throughout the volume, whereas a second leading edge (magenta) is found in between myelin layers (orange).

File name: Supplementary Movie 6

Description: 3D reconstruction from FIB-SEM of a P21 *Caspr*^-/-^*Caspr2*^-/-^*Mag*^-/-^ double myelinated axon. 3D reconstruction of a double myelinated axon with one leading edge from a 13 µm x 6 µm x 35 µm FIB-SEM volume. A second myelin segment (green) is found on top of myelin (orange) on an axon (blue).

File name: Supplementary Movie 7

Description: 3D reconstruction from FIB-SEM of a P21 *Caspr*^-/-^*Caspr2*^-/-^*Mag*^-/-^ double myelinated axon with two leading edges. 3D reconstruction of a double myelinated axon with one leading edge from a 13 µm x 6 µm x 35 µm FIB-SEM volume. One leading edge (green) is in contact with the axon (blue) throughout the volume, whereas a second leading edge (magenta) is found in between myelin layers (orange).
