# Supplementary material for: Two adhesive systems cooperatively regulate axon ensheathment and myelin growth in the CNS
Source: Nat Commun. 2019 Oct 22;10:4794. doi: 10.1038/s41467-019-12789-z (PMC6805957; doi:10.1038/s41467-019-12789-z)
Supplement: Supplementary file 2 — Peer Review [file 41467_2019_12789_MOESM2_ESM.pdf]

Reviewers' comments:

Reviewer #1 (Remarks to the Author):

The manuscript by Djannatian et al., describes the role of two types of adhesion in regulating targeting and myelination in the CNS. Using zebrafish and mice, the authors attempt to demonstrate that paranodal and internodal adhesion function synergistically to regulate axonal recognition and proper targeting of myelin. Overall, the phenotypes observed are intriguing (albeit descriptive in nature), however, the conclusions are a bit overstated and additional data is necessary to fully complete the story.

Major concerns:

1. The term mistargeting is not accurate for the myelin overgrowth (abstract). This term may represent the aberrant cell body wrapping, but the "double myelin" phenotype is not a targeting issue.

2. Without EM data, the "myelinated cell bodies" in the zebrafish is also an inaccurate statement and may just represent ensheathment. Additionally, do the single mutants (cntn1b-/-, caspr-/-, nfscb-/-) display aberrant cell body ensheathment in mice? Do the single mutants in zebrafish exhibit any double myelination? This is also never examined. Do the double mutants (cntn1b-/-; mag-/-, caspr-/-; mag-/-) display the cell body ensheathment phenotype? It is strange that this data is omitted—as it may help illustrate the differences found in the mutants and between zebrafish and mice. This is also true for the shortened internodes. They are not presented for the mouse mutants and only quantified in the zebrafish.

3. If cell body ensheathment is not apparent in the double mutants but only in the single paranodal mutants, than cell body ensheathment requires mag expression. Indeed, this would be a strange finding and would require additional data.

4. The cooperation between paranodal adhesion and internodal adhesion is required for proper myelin growth and wrapping demonstrated by knocking out two different axonal paranodal adhesion proteins in combination with two different glial adhesion proteins. Does knockout of two paranodal or two internodal adhesion proteins result in similar effects?

5. The data presented concerning the role of sphingolipids contributing to the localization and clustering of Caspr is quite confusing and missing a rationale. Is the point to mislocalize Caspr and demonstrate "double myelin" when crossed to the mag -/- mice? If so, this experiment should be performed. Additionally, using ganglioside–deficient mice and treatment with fumonicin B1 alter sphingolipids and gangliosides globally. This data is tangential at best and doesn't help resolve any of the phenotypes observed in the mouse and fish mutants.

Reviewer #2 (Remarks to the Author):

The mechanisms by which the complex three-dimensional architecture of the myelin sheath is generated remains very poorly understood. This paper makes a very important contribution by showing the cooperative interaction of two sets of adhesion molecules, one at the ends of each forming sheath and the other along the leading edge, enables the wrapping and lengthening of the membranous extension that eventually forms the sheath. The results are convincingly shown using zebrafish and mice, and combination of genetic lockouts, optical imaging three-dimensional electron microscopy is very compelling. The work addresses a major question in the field and will be considerable interest.

I have three comments for improving the manuscript.

First, it is important to have a better understanding of the abnormalities of actin formation as a result of loss the adhesion systems. The image shown is very interesting, but very preliminary. Clear imaging of the leading edge in the double mutant and quantification of the changes in actin within this leading-edge would be important. To what extent is this leading edge loaded with actin in the absence of adhesion molecule locking?

Second, the sphingolipid story is interesting in its own right but it really doesn't fit well within the logic of this paper. I can't see, with the paper constructed in its present form, how its inclusion can be justified

Third, experiments on artificial fibres have shown that myelin sheaths with multi-lamellar compact structure can be formed in the absence of any adhesion signalling between the axon and the oligodendrocyte. I appreciate that we still have a very poor understanding of the three-dimensional structure of the sheaths on fibres, particularly at their ends, but this should at least be discussed as it might be otherwise used as an argument against the hypothesis presented here by those less aware of the shortcomings of the fibre data.

Reviewer #3 (Remarks to the Author):

This is a very interesting and important paper that make a substantial contribution to our understanding of one of the most complex and mysterious processes in cell biology - the formation of the myelin sheath. Using both zebrafish and mouse models the authors provide a very persuasive case for a model (nicely summarised in figure 7) of a process which involves adhesion of the myelinating oligodendrocyte leading edge to the axon while, in a coordinated way, anchoring of the newly formed loops at the paranode. The experiments have been conducted to a very high standard and the conclusions drawn are entirely supported by the data presented in a very strong series of figures. I have no major concerns regarding this paper, which will make a significant contribution to the field.

Minor comments

Line 27 - 'use' rather than 'using'?

Line 102 - does the data really show that these three molecules form the axo-glial junction? They may well do, but this is not the data that proves that.

Line 112/127 - 'ataxic'?

Line 216 - 'abnormal' rather than 'degenerated' perhaps?

Reviewer #4 (Remarks to the Author):

How myelin producing cells orchestrate the growth and organisation of this complex material is poorly understood. Proper regulation of myelin structure is essential for neural function and behaviour, improving our understanding has to how complex glial axon interactions may regulate structural organisation of this substrate is of wide interest to the broader neuroscience field.

Djannatian et al., put forward a new perspective that MAG and the paranodal adhesion molecules work

synergistically to guide myelin sheath growth in a conserved mechanism across vertebrate and mammalian systems. While other work has identified axon-glial interactions are important for regulating myelination, the perspective put forward by Djannatian et al., is novel and would be of significant interest to the wider neuroscience community.

Overall the article is well written, the molecular interactions are complex, and the authors have done a good job of explaining the material in a clear and digestible manner. The authors have gone to some effort in using appropriate imaging techniques to try and to improve our understating of how complex 3D biological structures are altered when molecular interactions have been disrupted. I believe this work will have an impact on the way we think about myelin growth. Importantly, it provides good evidence that the process of orchestrating lipid into the complex structure that is myelin, is not simply a passive process, an idea this work will help in shaping.

I have several questions that if answered would strengthen the argument overall model. I do not see why this work would not be suitable for publication if these questions are addressed.

Major:
The evidence put forward supports the model that MAG interacting with gangliosides displaces axon adhesion molecules toward the respective lateral margins of the growing internode, while the paranodal adhesion molecules remain in tight association and shelter the growing edge leading edge. Loss of both systems disrupts this organisation and the leading edge becomes promiscuous leading to aberrant myelination.

All of the experiments are focused on the CNS, however peripheral nerves and schwann cells express the proteins disrupted in the knockout mice. Why is there no mention of, or any examination of a peripheral nerve / Shawn cell myelination phenotype? Whether this model is overarching is important, one would expect it to be observed in the process of peripheral nerve myelination growth as well? If not, some discussion as to why is required?
Essential: There should be some data included from the peripheral nerves of the knockout mouse lines used. I believe that this should be included as part of this paper, given that the genetic tools used are not directed exclusively to the CNS and that the model proposed could be applicable to the peripheral nervous system as well. If this model does not apply to peripheral nerve myelination that is fine, however it warrants investigation and discussion.

Minor
1) In terms of oligodendrocyte development in zebrafish, why do oligodendrocytes myelinate cell bodies in Wt animals initially? This statement is left with no follow up commentary. Are the adhesion molecules not expressed at these early stages? Also is the sporadic aberrant cell body myelination that persists due to newly made cells that initially myelinate incorrectly and then this process is resolved by the expression of the adhesion molecules.

2) This relates to the statistical representation of data on myelin sheath length depicted in Figure 2D average sheet length per genotype, clearly there is a spread change across the genetically distinct populations. However, I dislike the grouping of technical replicates across individuals for the stats. Why not just represent the average sheath length per animal? If induvial oligos have some long and some short sheaths, is the per animal effect still significant or washed out? I would like to see these statistics represented as a per animal average sheath length.

3) Again, relating to the cntn1b-/-mag-/- animals. Do the authors have a perspective on the overall growth of myelin or membrane production. This question relates to figure 2 F-H. The data is very clear that sheaths are not initially as long and are not elongating at the same rate. Is this reflective of a retardation of myelin growth or that the myelin is growing, but just folding on itself and thus the sheaths not elongating? More clarification on this issue is required to be included in the text.

4) Similar to the point in 3, in the caspr-/-mag-/-, do the authors think that the overall membrane growth per cells has decreased, less sheaths per oligo and less meylin area, but selectively increased in internodes where there is double sheath pathology? If this is so this should emphasized more clearly in the text.

5) Why is f-actin displaced in the mutants? What happens to the overall levels of f-actin? Has its overall levels been altered in these mutants this is important given its role in assembly / disassembly at the leading edge?

6) What is the authors perspective on why only some and not all internodes are impacted in the double knockouts? While many processes within the oligodendroglia of the mutant animals have aberrant myelination others remain intact? Some discussion around this point is required.

Is the pathology in the knockouts also in some way influenced by the diversity in the oligodendrocyte, and or the axon populations that they interact with? It is clear that oligodendrocyte heterogeneity exists across different white matter and grey matter structures, there is also neural diversity. Do the authors think that particular subclass of OLs with particular sub-classes of axons (neurons) would be more susceptible to undergoing aberrant myelination in the knockouts? Some discussion around this point is required.

Point-to-point response:

Reviewer #1

The manuscript by Djannatian et al., describes the role of two types of adhesion in regulating targeting and myelination in the CNS. Using zebrafish and mice, the authors attempt to demonstrate that paranodal and internodal adhesion function synergistically to regulate axonal recognition and proper targeting of myelin. Overall, the phenotypes observed are intriguing (albeit descriptive in nature), however, the conclusions are a bit overstated and additional data is necessary to fully complete the story.

We thank the reviewer for the positive comments and helpful suggestions.

Major concerns:

1. The term mistargeting is not accurate for the myelin overgrowth (abstract). This term may represent the aberrant cell body wrapping, but the "double myelin" phenotype is not a targeting issue.

We agree with the reviewer and now use the term misplacement throughout the manuscript.

2. Without EM data, the "myelinated cell bodies" in the zebrafish is also an inaccurate statement and may just represent ensheathment. Additionally, do the single mutants (cntn1b-/-, caspr-/-, nfscb-/-) display aberrant cell body ensheathment in mice? Do the single mutants in zebrafish exhibit any double myelination? This is also never examined. Do the double mutants (cntn1b-/-; mag-/-, caspr-/-; mag-/-) display the cell body ensheathment phenotype? It is strange that this data is omitted—as it may help illustrate the differences found in the mutants and between zebrafish and mice. This is also true for the shortened internodes. They are not presented for the mouse mutants and only quantified in the zebrafish.

We have now addressed all of the above questions. 1) The term myelinated cell body has been replaced by cell body ensheathment as suggested by the reviewer. 2) Cntn1 KO mice do not show any cell body ensheathment in mice. This data was previously only mentioned in the discussion. We have now added these data to Supplementary Figure 6. 3) We analyzed single mutants in zebrafish and detected only very few double myelin sheaths. This data has been added to Supplementary Figure 3a. This data is consistent with our findings in mice showing that only the combined deletion of two adhesive systems results in high levels of double

myelination. 4) We have analyzed double mutants (cntn1b-/-; mag-/-, caspr-/-; mag-/-) in zebrafish and find cell body ensheathments as described in the single mutants. This data has been added to Supplementary Figure 2f,g. 5) The shortened internode in double mutants are quantified in mice and shown in Figure 5i,j.

3. If cell body ensheathment is not apparent in the double mutants but only in the single paranodal mutants, than cell body ensheathment requires mag expression. Indeed, this would be a strange finding and would require additional data.

As described above we also find cell body ensheathment in double mutants (cntn1b-/-; mag-/-, caspr-/-; mag-/-).

4. The cooperation between paranodal adhesion and internodal adhesion is required for proper myelin growth and wrapping demonstrated by knocking out two different axonal paranodal adhesion proteins in combination with two different glial adhesion proteins. Does knockout of two paranodal or two internodal adhesion proteins result in similar effects?

We have now also knocked out two paranodal adhesion molecules in combination in zebrafish (cntn1b-/-, nfascb-/-). We find that double mutants have similar myelin sheath lengths as single mutants and no significant increase in double myelin sheaths. There is a slight increase in cell body ensheathments. These data has been added to Supplementary Figure 2j-m.

5. The data presented concerning the role of sphingolipids contributing to the localization and clustering of Caspr is quite confusing and missing a rationale. Is the point to mislocalize Caspr and demonstrate “double myelin” when crossed to the mag -/- mice? If so, this experiment should be performed. Additionally, using ganglioside–deficient mice and treatment with fumonicin B1 alter sphingolipids and gangliosides globally. This data is tangential at best and doesn’t help resolve any of the phenotypes observed in the mouse and fish mutants.

A similar issue was raised by Reviewer 2. We follow the suggestion of both reviewers to remove the data from the manuscript as it doesn’t help to resolve any of the phenotypes.

Reviewer #2

The mechanisms by which the complex three-dimensional architecture of the myelin sheath is generated remains very poorly understood. This paper makes a very important contribution by showing the cooperative interaction of two sets of adhesion molecules, one at the ends of each forming sheath and the other along the leading edge, enables the wrapping and lengthening of the membranous extension that eventually forms the sheath. The results are convincingly shown using zebrafish and mice, and combination of genetic lockouts, optical imaging three-dimensional electron microscopy is very compelling. The work addresses a major question in the field and will be considerable interest.

We thank the reviewer for the supportive feedback and helpful comments.

I have three comments for improving the manuscript.

First, it is important to have a better understanding of the abnormalities of actin formation as a result of loss the adhesion systems. The image shown is very interesting, but very preliminary. Clear imaging of the leading edge in the double mutant and quantification of the changes in actin within this leading-edge would be important. To what extent is this leading edge loaded with actin in the absence of adhesion molecule locking?

We have now extended and improved the visualization of the leading edge using Lifeact-RFP. We have characterized the different stages of how Lifeact-RFP distributes during myelin formation. In wild-type fish Lifeact-RFP forms a thin spiral line along the internode as the internode elongates. When myelin elongation is terminated Lifeact-RFP largely disappears from the internode. A completely different picture was observed when Lifeact-RFP was expressed in *cntn1b-/-mag-/-* mutants. We found that Lifeact-RFP forms a thin spiral line at the earliest stages of myelination at 3 dpf. Later at 4 dpf, Lifeact-RFP forms a barbell-shaped structure with accumulation within the lateral edges of the elongating sheath. Furthermore, at 7 dpf, Lifeact-RFP failed to clear away from the myelin when sheath elongation was terminated. We have quantified the changes of Lifeact-RFP distribution between wild-type and mutant at 3 and 4 dpf (Figure 3d). We also determined the area occupied by Lifeact-RFP within the myelin sheath (EGFP-CAAX) and find that mutants accumulate increased amounts

of F-actin (Figure 3f). Together, these data provide clear evidence that the leading is severely disturbed in the absence of these adhesion molecules. Representative images and a quantitative analysis of these data are shown in a new Figure 3.

Second, the sphingolipid story is interesting in its own right but it really doesn't fit well within the logic of this paper. I can't see, with the paper constructed in its present form, how its inclusion can be justified.

A similar issue was raised by Reviewer 1. We follow the suggestion of both reviewers to remove the data from the manuscript as it doesn’t help to resolve any of the phenotypes.

Third, experiments on artificial fibres have shown that myelin sheaths with multi-lamellar compact structure can be formed in the absence of any adhesion signalling between the axon and the oligodendrocyte. I appreciate that we still have a very poor understanding of the three-dimensional structure of the sheaths on fibres, particularly at their ends, but this should at least be discussed as it might be otherwise used as an argument against the hypothesis presented here by those less aware of the shortcomings of the fibre data.

We now discuss the experiments on myelination of artificial fibers in more detail (page 16-17 in the discussion).

Reviewer #3

This is a very interesting and important paper that make a substantial contribution to our understanding of one of the most complex and mysterious processes in cell biology - the formation of the myelin sheath. Using both zebrafish and mouse models the authors provide a very persuasive case for a model (nicely summarised in figure 7) of a process which involves adhesion of the myelinating oligodendrocyte leading edge to the axon while, in a coordinated way, anchoring of the newly formed loops at the paranode. The experiments have been conducted to a very high standard and the conclusions drawn are entirely supported by the data presented in a very strong series of figures. I have no major concerns regarding this paper, which will make a significant contribution to the field.

We thank the reviewer for the positive comments on our manuscript.

Minor comments

Line 27 - ‘use’ rather than ‘using’?

Corrected

Line 102 - does the data really show that these three molecules form the axo-glial junction? They may well do, but this is not the data that proves that.

Corrected

Line 112/127 - ‘ataxic’?

Corrected

Line 216 - ‘abnormal’ rather than ‘degenerated’ perhaps?

Corrected

Reviewer #4

How myelin producing cells orchestrate the growth and organisation of this complex material is poorly understood. Proper regulation of myelin structure is essential for neural function and behaviour, improving our understanding has to how complex glial axon interactions may regulate structural organisation of this substrate is of wide interest to the broader neuroscience field.

Djannatian et al., put forward a new perspective that MAG and the paranodal adhesion molecules work synergistically to guide myelin sheath growth in a conserved mechanism across vertebrate and mammalian systems. While other work has identified axon-glial interactions are important for regulating myelination, the perspective put forward by

Djannatian et al., is novel and would be of significant interest to the wider neuroscience community.

Overall the article is well written, the molecular interactions are complex, and the authors have done a good job of explaining the material in a clear and digestible manner. The authors have gone to some effort in using appropriate imaging techniques to try and to improve our understating of how complex 3D biological structures are altered when molecular interactions have been disrupted. I believe this work will have an impact on the way we think about myelin growth. Importantly, it provides good evidence that the process of orchestrating lipid into the complex structure that is myelin, is not simply a passive process, an idea this work will help in shaping.

I have several questions that if answered would strengthen the argument overall model. I do not see why this work would not be suitable for publication if these questions are addressed.

We thank the reviewer for the positive comments and helpful suggestions.

Major:

The evidence put forward supports the model that MAG interacting with gangliosides displaces axon adhesion molecules toward the respective lateral margins of the growing internode, while the paranodal adhesion molecules remain in tight association and shelter the growing edge leading edge. Loss of both systems disrupts this organisation and the leading edge becomes promiscuous leading to aberrant myelination.

All of the experiments are focused on the CNS, however peripheral nerves and schwann cells express the proteins disrupted in the knockout mice. Why is there no mention of, or any examination of a peripheral nerve / Shawn cell myelination phenotype? Whether this model is overarching is important, one would expect it to be observed in the process of peripheral nerve myelination growth as well? If not, some discussion as to why is required? Essential: There should be some data included from the peripheral nerves of the knockout mouse lines used. I believe that this should be included as part of this paper, given that the genetic tools used are not directed exclusively to the CNS and that the model proposed could

be applicable to the peripheral nervous system as well. If this model does not apply to peripheral nerve myelination that is fine, however it warrants investigation and discussion.

We have now analyzed cross-sections of peripheral sciatic nerves of Caspr and Mag double mutant mice by electron microscopy. In contrast to the CNS, large amounts of double myelin sheaths are not observed in the PNS. Quantification shows that the number of double myelin sheaths is not different between double mutants and wild-type controls. Thus, the role of adhesion molecules differs between the peripheral and central nervous system. We have added the data to a Supplementary Figure 7 and discuss the findings on page 12 and page 17.

Minor

1) In terms of oligodendrocyte development in zebrafish, why do oligodendrocytes myelinate cell bodies in Wt animals initially? This statement is left with no follow up commentary. Are the adhesion molecules not expressed at these early stages? Also is the sporadic aberrant cell body myelination that persists due to newly made cells that initially myelinate incorrectly and then this process is resolved by the expression of the adhesion molecules.

We do not know why oligodendrocyes also ensheath cell bodies initially, but we believe that developmental myelination is not perfect and requires “pruning”. In fact, we are able to show that cell body ensheathments are retracted in wild-type fish clearing away the mistargeted sheaths. We agree with the reviewer that this is an interesting topic that we now also discuss on page 16/17.

2) This relates to the statistical representation of data on myelin sheath length depicted in Figure 2D average sheet length per genotype, clearly there is a spread change across the genetically distinct populations. However, I dislike the grouping of technical replicates across individuals for the stats. Why not just represent the average sheath length per animal? If induvial oligos have some long and some short sheaths, is the per animal effect still significant or washed out? I would like to see these statistics represented as a per animal average sheath length.

We followed the reviewer’s suggestion and now present the sheath length per genotype per animal. The significance of the data remains and is shown in Figure 2d.

3) Again, relating to the cntn1b-/-mag-/- animals. Do the authors have a perspective on the overall growth of myelin or membrane production. This question relates to figure 2 F-H. The data is very clear that sheaths are not initially as long and are not elongating at the same rate. Is this reflective of a retardation of myelin growth or that the myelin is growing, but just folding on itself and thus the sheaths not elongating? More clarification on this issue is required to be included in the text.

4) Similar to the point in 3, in the caspr-/-mag-/-, do the authors think that the overall membrane growth per cells has decreased, less sheaths per oligo and less meylin area, but selectively increased in internodes where there is double sheath pathology? If this is so this should emphasized more clearly in the text.

Combined answer to point 3 and 4: The reviewer raises an interesting issue. Cntn1b-/-mag-/- and caspr-/-mag-/- double mutants have shorter sheaths and less myelin on cross section, yet, double myelin sheaths are found. How is this explained? Our interpretation goes along the same line as suggested by the reviewer. We believe that in the absence of axo-glial adhesion molecules, the machinery of myelin membrane generation remains intact. Thus, in double mutant mice oligodendrocytes appear to produce myelin membrane efficiently. However, in the absence of adhesive strength the leading edge appears to be misguided and mislocated into the myelin sheaths and on top of other myelin sheaths. Yet, our data also indicate that oligodendrocytes in double mutant zebrafish generate fewer myelin sheaths per oligodendrocytes. It is possible that the traffic jam that is generated by the dysfunctional leading edge results in feed-back inhibition of myelin biogenesis. We now discuss these possibilities on page 16/17

5) Why is f-actin displaced in the mutants? What happens to the overall levels of f-actin? Has its overall levels been altered in these mutants this is important given its role in assembly / disassembly at the leading edge?

A similar issue was also raised by Reviewer 2. We have now extended our analysis on the actin cytoskeleton. We and others have previously provided evidence that a highly dynamic actin cytoskeleton (low F-actin levels) is required for myelin growth. We have characterized the different stages of how Lifeact-RFP distributes during myelin formation. In wild-type fish Lifeact-RFP forms a thin spiral line along the internode as the internode elongates. When

myelin elongation is terminated Lifeact-RFP largely disappears from the internode. A completely different picture was observed when Lifeact-RFP was expressed in *cntn1b-/-mag-/-* mutants. We found that Lifeact-RFP forms a thin spiral line at the earliest stages of myelination at 3 dpf. Later at 4 dpf, Lifeact-RFP forms a barbell-shaped structure with accumulation within the lateral edges of the elongating sheath. Furthermore, at 7 dpf, Lifeact-RFP failed to clear away from the myelin when sheath elongation was terminated. We have quantified the changes of Lifeact-RFP distribution between wild-type and mutant at 3 and 4 dpf (Figure 3d). We also determined the area occupied by Lifeact-RFP within the myelin sheath (EGFP-CAAX) and find that mutants accumulate increased amounts of F-actin (Figure 3f). Together, these data provide clear evidence that the leading is severely disturbed in the absence of these adhesion molecules. Representative images and a quantitative analysis of these data are shown in a new Figure 3.

6) What is the authors perspective on why only some and not all internodes are impacted in the double knockouts? While many processes within the oligodendroglia of the mutant animals have aberrant myelination others remain intact? Some discussion around this point is required.

On 2D cross section ~ a fifth of all myelin sheaths show aberrant myelin internodes with double myelin sheaths. When 3D EM using automated tape-collection ultramicrotome scanning electron microscopy (ATUM-SEM) was performed to visualize entire myelin internodes, we find that majority of internodes were affected. Only around 10% appeared normal. One reason why some internodes remain intact could be the slow growth of myelin in double mutants. We now discuss this point on page 20. Another reason could be heterogeneity of oligodendrocytes in their requirement of adhesion molecules as suggested below.

Is the pathology in the knockouts also in some way influenced by the diversity in the oligodendrocyte, and or the axon populations that they interact with? It is clear that oligodendrocyte heterogeneity exists across different white matter and grey matter structures, there is also neural diversity. Do the authors think that particular subclass of OLs with particular sub-classes of axons (neurons) would be more susceptible to undergoing aberrant myelination in the knockouts? Some discussion around this point is required

We now discuss this interesting possibility on page 16/17.

Reviewers' comments:

Reviewer #1 (Remarks to the Author):

The authors have dealt with most of the previous concerns by changes to text and additional experiments. However, the authors seem to have missed an opportunity to provide a cohesiveness to the manuscript.

1. The term mistargeting is now replaced by "misplacement". This is still not accurate. The underlying issue is whether the cell body wrapping and double myelin are the result of similar or competitive mechanisms. Is the aberrant cell body wrapping related at all to the double myelin? The double myelin is not "misplacement", but that the lateral overgrowth of membrane is not properly or spatially restricted. This becomes clear in comments 2 and 3.

2. Without EM data, it is impossible to know whether the cell body wrapping and double myelin are related in terms of mechanism. Replacing "myelinated cell body" with "ensheathment" is not accurate if the cell bodies are truly "myelinated" with compact wraps of membrane. EM analyses was previously shown in zebrafish and mice by the Lyons group. Why is this not performed?

While the Cntn KO mice do not show cell body ensheathment, do any of the other single KO mice show cell body ensheathment? This is important to understand if paranodal adhesion is limited to zebrafish only.

3. Looking at Supp Fig 2f and g, it becomes readily apparent that the single Caspr KO fish have more cell body wrapping than the double caspr/mag mutants. Does the presence of MAG encourage cell body wrapping in the absence of Caspr? Is this a demonstration of a competitive balance between cell body wrapping and double myelin? i.e. when double myelin occurs, fewer cell bodies are wrapped. In fact, it may be an inaccuarate statement to say that MAG is an internodal adhesive cue—especially as MAG is also localized to the paranode. This is an important point that needs to be addressed/discussed.

4. Addressed to my satisfaction

5. Addressed to my satisfaction

Reviewer #2 (Remarks to the Author):

This already strong paper has been improved further by attention to the reviewer's comments. The actin imaging is lovely. I have no further comments.

Reviewer #3 (Remarks to the Author):

The paper has been appropriately modified and strengthened.

Reviewer #4 (Remarks to the Author):

Djannatian et al., have demonstrated a concerted effort to address all of the major and minor questions put forward by this reviewer. The data presented provides new evidence that will shape thinking on how lipid membranes are orchestrated into the complex structure that is myelin. The data builds significantly on the existing literature detailing the complex axon-glial interactions required for

targeting and instructing proper myelination. Overall, I am satisfied with the revisions and believe this work to be suitable for publication.
I have listed my comments to each of the authors responses below:

Major:
The evidence put forward supports the model that MAG interacting with gangliosides displaces axon adhesion molecules toward the respective lateral margins of the growing internode, while the paranodal adhesion molecules remain in tight association and shelter the growing edge leading edge. Loss of both systems disrupts this organisation and the leading edge becomes promiscuous leading to aberrant myelination.
All of the experiments are focused on the CNS, however peripheral nerves and schwann cells express the proteins disrupted in the knockout mice. Why is there no mention of, or any examination of a peripheral nerve / Shawn cell myelination phenotype? Whether this model is overarching is important, one would expect it to be observed in the process of peripheral nerve myelination growth as well? If not, some discussion as to why is required? Essential: There should be some data included from the peripheral nerves of the knockout mouse lines used. I believe that this should be included as part of this paper, given that the genetic tools used are not directed exclusively to the CNS and that the model proposed could be applicable to the peripheral nervous system as well. If this model does not apply to peripheral nerve myelination that is fine, however it warrants investigation and discussion.

We have now analyzed cross-sections of peripheral sciatic nerves of Caspr and Mag double mutant mice by electron microscopy. In contrast to the CNS, large amounts of double myelin sheaths are not observed in the PNS. Quantification shows that the number of double myelin sheaths is not different between double mutants and wild-type controls. Thus, the role of adhesion molecules differs between the peripheral and central nervous system. We have added the data to a Supplementary Figure 7 and discuss the findings on page 12 and page 17.

This revision clearly informs that the mechanisms put forward are only at play for CNS oligodendrocyte-axon interactions and are not common to the peripheral nervous system. Schwann cells must either possess other mechanisms to compensate for the genetic perturbations, or these mechanisms are not at play in the development of peripheral myelin. The data demonstrates distinctions between the CNS and PNS that are important. I am satisfied with the authors response to this question.

Minor
1) In terms of oligodendrocyte development in zebrafish, why do oligodendrocytes myelinate cell bodies in Wt animals initially? This statement is left with no follow up commentary. Are the adhesion molecules not expressed at these early stages? Also is the sporadic aberrant cell body myelination that persists due to newly made cells that initially myelinate incorrectly and then this process is resolved by the expression of the adhesion molecules.

We do not know why oligodendrocyes also ensheath cell bodies initially, but we believe that developmental myelination is not perfect and requires "pruning". In fact, we are able to show that cell body ensheathments are retracted in wild-type fish clearing away the mistargeted sheaths. We agree with the reviewer that this is an interesting topic that we now also discuss on page 16/17.

I am satisfied with the discussion around this point.

2) This relates to the statistical representation of data on myelin sheath length depicted in Figure 2D average sheet length per genotype, clearly there is a spread change across the genetically distinct populations. However, I dislike the grouping of technical replicates across individuals for the stats. Why not just represent the average sheath length per animal? If induvial oligos have some long and some short sheaths, is the per animal effect still significant or washed out? I would like to see these statistics represented as a per animal average sheath length.

We followed the reviewer's suggestion and now present the sheath length per genotype per animal. The significance of the data remains and is shown in Figure 2d.
I am more confident with the statistical representation of the data and that how the tests have been conducted.

I am satisfied with the response to this question.

3) Again, relating to the cntn1b-/-mag-/- animals. Do the authors have a perspective on the overall growth of myelin or membrane production. This question relates to figure 2 F-H. The data is very clear that sheaths are not initially as long and are not elongating at the same rate. Is this reflective of a retardation of myelin growth or that the myelin is growing, but just folding on itself and thus the sheaths not elongating? More clarification on this issue is required to be included in the text.

4) Similar to the point in 3, in the caspr-/-mag-/-, do the authors think that the overall membrane growth per cells has decreased, less sheaths per oligo and less meylin area, but selectively increased in internodes where there is double sheath pathology? If this is so this should emphasized more clearly in the text.

Combined answer to point 3 and 4: The reviewer raises an interesting issue. Cntn1b-/-mag-/- and caspr-/-mag-/- double mutants have shorter sheaths and less myelin on cross section, yet, double myelin sheaths are found. How is this explained? Our interpretation goes along the same line as suggested by the reviewer. We believe that in the absence of axo-glial adhesion molecules, the machinery of myelin membrane generation remains intact. Thus, in double mutant mice oligodendrocytes appear to produce myelin membrane efficiently. However, in the absence of adhesive strength the leading edge appears to be misguided and mislocated into the myelin sheaths and on top of other myelin sheaths. Yet, our data also indicate that oligodendrocytes in double mutant zebrafish generate fewer myelin sheaths per oligodendrocytes. It is possible that the traffic jam that is generated by the dysfunctional leading-edge results in feed-back inhibition of myelin biogenesis. We now discuss these possibilities on page 16/17

Acknowledgement by the authors that it is still unclear as to how myelin biogenesis is ultimately affected is important, I am satisfied by the revised discussion to address this comment.

5) Why is f-actin displaced in the mutants? What happens to the overall levels of f-actin? Has its overall levels been altered in these mutants this is important given its role in assembly / disassembly at the leading edge?

A similar issue was also raised by Reviewer 2. We have now extended our analysis on the actin cytoskeleton. We and others have previously provided evidence that a highly dynamic actin cytoskeleton (low F-actin levels) is required for myelin growth. We have characterized the different stages of how Lifeact-RFP distributes during myelin formation. In wild-type fish Lifeact-RFP forms a thin spiral line along the internode as the internode elongates. When myelin elongation is terminated Lifeact-RFP largely disappears from the internode. A completely different picture was observed when Lifeact-RFP was expressed in cntn1b-/-mag- /- mutants. We found that Lifeact-RFP forms a thin spiral line at the earliest stages of myelination at 3 dpf. Later at 4 dpf, Lifeact-RFP forms a barbell-shaped structure with accumulation within the lateral edges of the elongating sheath. Furthermore, at 7 dpf, Lifeact- RFP failed to clear away from the myelin when sheath elongation was terminated. We have quantified the changes of Lifeact-RFP distribution between wild-type and mutant at 3 and 4 dpf (Figure 3d). We also determined the area occupied by Lifeact-RFP within the myelin sheath (EGFP-CAAX) and find that mutants accumulate increased amounts of F-actin (Figure 3f). Together, these data provide clear evidence that the leading is severely disturbed in the absence of these adhesion molecules. Representative images and a quantitative analysis of these data are shown in a new Figure 3.

I am satisfied with this additional analysis, it is important to understand that both f-actin levels remain elevated (which is distinct from Wt) and that that the leading edge is significantly disrupted in the cntn1b-/-mag- /- mutants. I am satisfied with this response.

6) What is the authors perspective on why only some and not all internodes are impacted in the double knockouts? While many processes within the oligodendroglia of the mutant animals have aberrant myelination others remain intact? Some discussion around this point is required.

On 2D cross section ~ a fifth of all myelin sheaths show aberrant myelin internodes with double myelin sheaths. When 3D EM using automated tape-collection ultramicrotome scanning electron microscopy (ATUM-SEM) was performed to visualize entire myelin internodes, we find that majority of internodes were affected. Only around 10% appeared normal. One reason why some internodes remain intact could be the slow growth of myelin in double mutants. We now discuss this point on page 20. Another reason could be heterogeneity of oligodendrocytes in their requirement of adhesion molecules as suggested below.

It is important that readers know that some internodes are normal, and that there is acknowledgement by the the authors that it still remains to be determined as to why? I am satisfied with the authors response to this question.

Is the pathology in the knockouts also in some way influenced by the diversity in the oligodendrocyte, and or the axon populations that they interact with? It is clear that oligodendrocyte heterogeneity exists across different white matter and grey matter structures, there is also neural diversity. Do the authors think that particular subclass of OLs with particular sub-classes of axons (neurons) would be more susceptible to undergoing aberrant myelination in the knockouts? Some discussion around this point is required

We now discuss this interesting possibility on page 16/17.

I am satisfied with the authors response.

Point-to-point response:

Reviewer #1

The authors have dealt with most of the previous concerns by changes to text and additional experiments. However, the authors seem to have missed an opportunity to provide a cohesiveness to the manuscript.

1. The term mistargeting is now replaced by “misplacement”. This is still not accurate. The underlying issue is whether the cell body wrapping and double myelin are the result of similar or competitive mechanisms. Is the aberrant cell body wrapping related at all to the double myelin? The double myelin is not “misplacement”, but that the lateral overgrowth of membrane is not properly or spatially restricted. This becomes clear in comments 2 and 3.

We used the term “misplacement” to refer to deposition of myelin on to improper structures such as on top of each other. To be more accurate we now use the term “unrestricted” myelin growth.

2. Without EM data, it is impossible to know whether the cell body wrapping and double myelin are related in terms of mechanism. Replacing “myelinated cell body” with “ensheathment” is not accurate if the cell bodies are truly “myelinated” with compact wraps of membrane. EM analyses was previously shown in zebrafish and mice by the Lyons group. Why is this not performed?

We apologize for omitting the EM analyses of cell bodies. We have done extensive EM on cell bodies of *cntn1b* mutants (n=3 fish at 10 dpf) using ATUM resulting in 500 different adjacent 200 nm sections used for SEM imaging. Our analyses show that there are one or more layers of membrane around the cell bodies. To be more accurate we now refer to these structures as cell body wrappings. This term includes both ensheathments and myelinated cell bodies. The data is shown in Figure 1e.

While the Cntn KO mice do not show cell body ensheathment, do any of the other single KO mice show cell body ensheathment? This is important to understand if paranodal adhesion is limited to zebrafish only.

We also analyzed MAG KO mice, which do not show cell body wrappings. This data has been added to Supplementary Figure 6.

3. Looking at Supp Fig 2f and g, it becomes readily apparent that the single Caspr KO fish have more cell body wrapping than the double caspr/mag mutants. Does the presence of MAG encourage cell body wrapping in the absence of Caspr? Is this a demonstration of a competitive balance between cell body wrapping and double myelin? i.e. when double myelin occurs, fewer cell bodies are wrapped. In fact, it may be an inaccuarate statement to say that

MAG is an internodal adhesive cue—especially as MAG is also localized to the paranode. This is an important point that needs to be addressed/discussed.

As correctly noted by the reviewer cell body wrappings are reduced in *caspr-/-mag-/-* as compared to the single *caspr* mutants. Cell body wrappings occur when production of myelin is high (Almeida et al. 2018). Thus, the reduction of myelin generation in *caspr-/-mag-/-* may explain the relative decrease in cell body wrapping. A competitive balance between cell body wrappings and double myelination is another explanation. To be more accurate we now refer to MAG as a paranodal/internodal protein. These points have now been added and are discussed on page 16 and 18 (marked in yellow).

REVIEWERS' COMMENTS:

Reviewer #1 (Remarks to the Author):

The authors have addressed all of the major concerns.

Point-to-point response:

Reviewer 1: The authors have addressed all of the major concerns

We thank the reviewer for the helpful suggestions
